# Extraction of Membrane Components from *Neisseria gonorrhoeae* Using Catanionic Surfactant Vesicles: A New Approach for the Study of Bacterial Surface Molecules

**DOI:** 10.3390/pharmaceutics12090787

**Published:** 2020-08-20

**Authors:** Daniel C. Stein, Lenea H. Stocker, Abigail E. Powell, Salsawi Kebede, David Watts, Emma Williams, Nicholas Soto, Avantika Dhabaria, Catherine Fenselau, Shweta Ganapati, Philip DeShong

**Affiliations:** 1 Department of Cell Biology and Molecular Genetics, University of Maryland, College Park, MD 20742, USA; rassalsawi@gmail.com (S.K.); emwill224@gmail.com (E.W.); 2 Department of Chemistry and Biochemistry, University of Maryland, College Park, MD 20742, USA; lenea.h.stocker@gmail.com (L.H.S.); aepowell@stanford.edu (A.E.P.); nsoto1@terpmail.umd.edu (N.S.); avantika.dhabaria@gmail.com (A.D.); fenselau@umd.edu (C.F.); deshong@umd.edu (P.D.); 3 SD Nanosciences, College Park, MD 20742, USA; dwatts12@umd.edu (D.W.); shweta.ganapati@gmail.com (S.G.)

**Keywords:** catanionic surfactant vesicles, antigen, vaccine, *Neisseria gonorrhoeae*, mass spectral analysis

## Abstract

Identification of antigens is important for vaccine production. We tested extraction protocols using cetyltrimethylammonium tosylate (CTAT) and sodium dodecylbenzenesulfonate (SDBS) to formulate surfactant vesicles (SVs) containing components from *Neisseria gonorrhoeae*. Carbohydrate and protein assays demonstrated that protein and carbohydrates were incorporated into the vesicle leaflet. Depending on the extraction protocol utilized, 100–400 µg of protein/mL of SVs solution was obtained. Gel electrophoresis followed by silver staining demonstrated that SV extracts contained lipooligosaccharide and a subset of bacterial proteins and lipoproteins. Western blotting and mass spectral analysis indicated that the majority of the proteins were derived from the outer membrane. Mass spectrometric and bioinformatics analysis of SVs identified 29 membrane proteins, including porin and opacity-associated protein. Proteins embedded in the SVs leaflet could be degraded by the addition of trypsin or proteinase K. Our data showed that the incorporation of CTAT and SDBS into vesicles eliminated their toxicity as measured by a THP-1 killing assay. Incorporation of gonococcal cell surface components into SVs reduced toxicity as compared to the whole cell extracts, as measured by cytokine induction, while retaining the immunogenicity. This process constitutes a general method for extracting bacterial surface components and identification of antigens that might be included in vaccines.

## 1. Introduction

Cell surface receptors play a crucial role in cell–cell communication and recognition [1]. In Gram-negative bacteria, bacterial cell-host cell recognition is a key feature of pathogen virulence [2] and in vivo biofilm formation [3]. Due to the complexity of biological membranes, these interactions are often studied using artificial membranes, most often in liposomes, in order to determine the specific role of membrane components or their mechanism of action [4]. Despite the success of using liposomes to formulate cell surface components in the laboratory, they have significant limitations as vehicles for displaying cell surface proteins and lipids in a stable membrane-like environment for biochemical studies or in clinically relevant vaccine formulations (see Akbarzadeh et al. for a review of liposomes) [5].

Studies in our labs have focused on exploiting the inherent qualities of catanionic surfactant vesicles (SVs) for the production of functionalized nanomaterials by the incorporation of biological components, such as proteins, complex carbohydrates, and nucleic acid derivatives into SVs [6,7]. SVs have numerous advantages over their liposomal counterparts as a vehicle for extraction and presentation of cell surface components in vaccine formulations and for studies on membrane biology. The ability to create complex nanomaterials teamed with the robust character of SVs, particularly in biological buffers, would make the resulting functionalized vesicles attractive for vaccine formulations and drug delivery due to facilitate preparation, ease of handling, and stability in long-term storage. Methods that improve the extraction of bacterial antigens from membranes or simplify the reconstitution of cellular components into stable hydrophobic matrices would represent a major improvement in understanding how various bacterial surface components contribute to bacterial biology.

In this paper, we describe protocols for the extraction of cell surface components from the Gram-negative bacterial pathogen *N. gonorrhoeae* directly into the leaflet of SVs. Mass spectral, immunological, and bioinformatic analysis of the proteins contained within the SV extracts demonstrate that key cell surface components are effectively incorporated into the leaflet of SVs. We determined several biological parameters associated with SVs (toxicity and immunostimulatory properties) and showed that the resulting functionalized SVs can induce an immune response similar to what is seen with whole cell extracts but with reduced toxicity. The resulting cell surface-functionalized SVs serve as an “artificial pathogen”, which can be employed in the identification and immunological and biological characterization of bacterial surface components.

## 2. Materials and Methods

### 2.1. Materials General

All chemicals and solvents were purchased from commercial suppliers and were used as received unless otherwise noted. All aqueous solutions were prepared using water from a Millipore (18 MΩ) water purification system (MilliporeSigma, Burlington, MA, USA). An Ocean Optics USB 2000 Spectrometer (Ocean Insight, Largo, FL, USA) was used to measure UV-VIS absorbance of samples.

### 2.2. Cell Cultures

*N. gonorrhoeae* F62ΔlgtD [8] was grown in GCP broth [9] for 48 h to an OD of ~1.0 (650 nm) (10^9^ CFU/mL). Aliquots (2 mL) of bacterial cell culture were collected by centrifugation at 9000 RPM in a Sorvall SS34 rotor (ThermoFisher, Waltham, MA, USA) for 5 min, the supernatant decanted, and cell pellets stored at −20 °C until needed.

### 2.3. Surfactant Vesicle Preparation

SVs prepared in deionized water were used for initial studies assessing overall protein and carbohydrate incorporation as well as protein identification. Sodium dodecylbenzenesulfonate (SDBS) was purchased from TCI America (Portland, OR, USA) and was utilized without further purification. Cetyltrimethylammonium tosylate (CTAT) was purchased from Sigma (St. Louis, MO USA) and recrystallized from ethanol–acetone. The purified solid was stored at room temperature in a desiccator containing Drierite. Five different protocols were used to prepare neisserial vesicles. For all methods, the ratio of the two surfactants was always 70 mg of SDBS to 30 mg of CTAT in 10 mL of water. For method (1) solid surfactants were added to the bacterial cell pellet followed by the addition of water; for method (2) equal volumes of an aqueous SDBS solution added to the bacterial cell pellet followed by the addition of an aqueous CTAT solution; for method (3) an aqueous CTAT solution was added to the bacterial cell pellet followed by the addition of solid SDBS; for method (4) an aqueous SDBS solution was added to bacterial cell pellet followed by the addition of solid CTAT; for method (5) SVs were formulated in water and then added to the bacterial cell pellet. For each method, the mixture was stirred using a magnetic stir bar for 18 h. The resulting suspensions were centrifuged for 5 min at 5000 RPM in an SS34 rotor and the supernatant collected and saved. SVs do not pellet under these centrifugation conditions, while bacteria and bacterial-derived cell debris are pelleted. The resulting colloidal supernatant, milky in appearance, was purified by gel filtration through a column containing Sephadex G-100, where a 1.0 mL aliquot of vesicle extract solution was added to a column (length 5.5 cm, diameter 1.5 cm). Vesicles were eluted with water and 1.0 mL aliquots collected, yielding fourteen fractions.

Fractions were characterized for the presence of carbohydrate using a phenol–sulfuric colorimetric assay [6]. Vesicles were analyzed for the presence of protein using a modified procedure of the Pierce bicinchoninic acid (BCA) assay [10]. For the modified BCA assay, the working reagent was prepared using a 50:1 *v*/*v* ratio of Reagent A (sodium carbonate, sodium bicarbonate, bicinchoninic acid, and sodium tartrate in 0.1 M sodium hydroxide) to Reagent B (4% copper (II) sulfate). The test-tube protocol was used in which 2.0 mL of working reagent was added to 0.1 mL of the extraction sample, sealed, and heated at 60 °C for 30 min. The samples were then cooled for 10 min at −20 °C to stop the reaction. To prevent intact vesicles from scattering light and interfering with the absorbance of samples, each sample was diluted with an equal volume of ethanol before measurement. The absorbance was measured at 562 nm and compared against a standard curve created using a known quantity of bovine serum albumin.

### 2.4. Vaccine Preparations

SVs were prepared under sterile conditions for biological assays including cytotoxicity, cytokine induction, and immunogenicity assays. *N. gonorrhoeae* F62ΔlgtD [8] was grown up as outlined above, the culture pelleted via centrifugation to give a large pellet with a wet weight of 2 g. The pellet was resuspended in 50 mL PBS 1X and aliquoted into 10 samples, each with ~200 mg wet weight. The pellets were pasteurized at 65 °C for 1 h in a temperature-controlled water bath. Pellets were stored at −20 °C until needed.

To prepare the vesicles for vaccine, one of the aliquots was thawed and resuspended in 30 mL sterile PBS and placed in a sonicating bath for 3 h with intermittent vortexing to achieve homogeneity. 15 mL was transferred from the suspension to a vial containing a Teflon coated stir bar and 90 mg SDBS, which had previously been autoclaved. After stirring at room temperature for 1 h, the mixture along with the stir bar was transferred to a second vial, which contained 60 mg CTAT and stirred for 18 h. The next day, the milky white mixture was transferred back to a conical tube and centrifuged at 6000 rpm on a 14 cm radius rotor (5630 g) for 10 min. The supernatant was removed and passed through a sterile 45 µm cellulose acetate syringe filter. This solution is stable for months at 4 °C and was stored until needed. The crude extract (1.0 mL) was removed and purified by gel filtration through a sterile column (length 5.5 cm, diameter 1.5 cm) containing autoclaved Sepharose^®^ (Sigma, St. Louis, MO, USA) CL-2B gel. The fractions containing SVs eluting from 3.0–4.5 mL (1.5 mL total volume) were collected and used without further purification. These SVs contained ~90 µg/mL of protein, as measured by BCA.

The whole cell extract vaccine (WCE) was prepared by suspending a ~200 mg of *N. gonorrhoeae* F62ΔlgtD (identical to that used to prepare sterile SVs) in 25 mL sterile PBS, boiled for 1 h and then transferred to a sonicating bath for 24 h to achieve homogeneity. The WCE was stored at 4 °C until needed. BCA analysis revealed a protein content of 220 µg/mL. Aliquots for use in biological assays were diluted so that the protein content matched that of the SV vaccines.

### 2.5. Gel Electrophoresis

Purified vesicle samples from extractions of *N. gonorrhoeae* and a molecular weight standard were mixed with loading buffer (5X SDS–PAGE gel loading buffer: 0.25 M Tris–HCl, pH 6.8, 15% SDS, 50% glycerol, 25% β-mercaptoethanol, and 0.01% bromophenol blue) and boiled for 10 min. The molecular weight standard was sometimes spiked with 0.5 µg of LOS purified from F62ΔlgtD. Samples were loaded onto an SDS–Tricine polyacrylamide gel (16.5%) using Tris–Tricine as the running buffer (BioRad, Philadelphia, PA, USA) and run for ~4.5 h at 100 V on ice (Bio-Rad Model 200/2.0 power supply, (BioRad, Philadelphia, PA, USA) or onto an SDS–Glycine polyacrylamide gel (12%) using Tris–glycine as the running buffer (BioRad) and run for ~1 h at 100 V on ice.

### 2.6. Silver Staining

After electrophoresis, the gels were incubated in a fixing solution (500 mL of 38% ethanol and 25 mL glacial acetic acid) overnight on a shaker at room temperature. Gels were silver stained according to a modified procedure [11]. The gel was transferred to 100 mL of an aqueous periodic acid (0.036 M) wash for 5 min and then rinsed four times with water for 30 min with gentle shaking. The silver staining solution was prepared by adding 4.0 mL of diluted silver nitrate (4.7 mmol) dropwise to Solution 1 (1 pellet sodium hydroxide, 25 mL water, and 1.40 mL of 30% ammonium hydroxide). The silver staining solution was brought to a final volume of 100 mL with water, added to a tray containing the gel, with incubation for 15 min with gentle shaking at room temperature. After staining, the gel was washed with water six times (15 min each) and incubated in a developing solution (95 μL formaldehyde 37% solution, 1 mL citric acid 25 mg/mL, and 500 mL water) until bands became visible. The gel was washed in water and then imaged.

### 2.7. Western Blotting

Western blotting was performed by transferring proteins and carbohydrates from SDS–PAGE gels to a nylon membrane under standard conditions [12]. The membrane was rinsed with a PBS/Tween-20/non-fat dried milk solution 5 times for 15 min each and then incubated with anti-gonococcal polyclonal antisera (mouse or goat) [13], diluted in a blocking solution of 2% casein [14], and incubated with gentle shaking for 2 h. The membrane was rinsed with a PBS/Tween-20 solution five times for 15 min each and then incubated with donkey anti-goat or mouse HRP (Jackson ImmunoResearch laboratories Inc., West Grove, PA, USA) (1:100,000) secondary antibody solution prepared in 2% casein, with incubation at room temperature with shaking for 2 h. The membrane was rinsed with a PBS/Tween-20 solution five times for 15 min each and then incubated with Western blotting chemiluminescence solution (PerkinElmer, Waltham, MA, USA) and the image was captured on a Fuji LAS3000 image analysis system.

### 2.8. Surfactant Vesicle (SV) Protection Experiments

Vesicle-containing fractions were digested by adding 10 μL proteinase K (25 mg/mL) to a 500 μL sample, with incubation at 65 °C or by adding 10 μL trypsin (25 mg/mL) to a 500 μL sample, with incubation at 37 °C. A control cell pellet extract was prepared by suspending the pellet in 1.0 mL of water.

### 2.9. Proteomics Analysis

Vesicle extracts were prepared from cell pellets formed from 20 mL, 40 mL, or 60 mL of cells. Known amounts of protein were spotted from these preparations in each lane of a one-dimensional gel (Tris–HCl, 8–16% gradient). Whole cell lysate was spotted in a fourth lane. The gel was developed and stained with Coomassie blue stain (Sigma, St. Louis, MO USA).

Fifteen slices were cut from each lane and subjected to overnight in-gel tryptic digestion (13 ng/μL) using a standard procedure [15]. The resulting peptides were extracted and injected into a capLC–MS/MS LTQ–orbitrap (ThermoFisher, San Jose, CA, USA), as described elsewhere [16]. Peptide and protein candidates were analyzed using the search program MASCOT 2.3 (Matrix Science, London, UK), and protein identifications were based on the number of associated tryptic peptides and the reliability of the peptide identifications. Protein databases were searched, one compiled of all *Neisseria* sequences from NCBInr and one comprised from only the proteins in NCBInr from the species *N. gonorrhoeae* (www.ncbi.nlm.nih.gov.com).

### 2.10. Dynamic Light Scattering (DLS) Analysis

Dynamic light scattering (DLS) experiments were carried out on a Malvern Nano ZS90 instrument (Malvern Panalytical Ltd., Malvern, WR14 1XZ, United Kingdom) with a 532 nm laser and scattering angle of 90°. Disposable polystyrene cuvettes were used with a sample volume between 1.0 and 1.5 mL. The laser was passed through an auto attenuator, so that the intensity of the scattered light was between 200 and 400 kcps for every sample. Each sample was measured twice, and each measurement was an average of at least 10 individual runs. All samples were found to give unimodal scattering data, and thus, the reported size (Z-avg) and polydispersity index (PDI), derived from the cumulants method, is considered to represent the true average hydrodynamic radius of the particles.

### 2.11. Cytotoxicity Assays

THP-1 cells were obtained from the American Type Culture Collection (ATCC) (Manassas, VA, USA) and grown in RPMI1640 Media supplemented with 10% FBS (no antibiotics). When cells reached sufficient density, they were diluted to 5 × 10^4^ cells/mL with complete medium plus 16 nM (10 ng/mL) phorbol myristate acetate (PMA). Aliquots (200 µL) were added to wells in a sterile 96-well plate and incubated at 37 °C for 24 h. After 24 h, the media was carefully aspirated from each well. Each well was then replaced with a complete medium plus various dilutions of the vesicle or vesicle components. Sodium azide (1% final concentration) was used as a positive killing control. After 24 h, Alamar Blue (Biorad, Philadelphia, PA, USA) was added to each well to 10% of the well volume (20 µL). The plate was then wrapped in aluminum foil to exclude light and incubated at 37 °C for 3 h. Absorbance was determined, and the reading of the untreated well was set to 100% viability.

### 2.12. Cytokine Induction Assays

Human THP-1 cells were grown in RPMI-1640 supplemented with 10% FBS (no antibiotics). Cells were diluted to a final concentration of 2 × 10^5^ cells in 500 µL of complete media +10 ng/mL PMA (phorbol 12-myristate 13-acetate) in a 48-well cell culture plate and incubated in 37 °C for 24 h. After 24 h, 50 µL of each test sample was added to the wells, and cells were further incubated for 24 h under the same conditions. After 24 h incubation, broth from each well was collected separately and centrifuged to collect supernatants. Collected supernatants were stored at −80 °C until the cytokine assays were performed. Invitrogen Human Cytokine Elisa Kits (Invotrogen, Carlsbad, CA, USA) were used to measure cytokine levels in collected supernatants, according to the manufacturer’s instructions.

### 2.13. Immunization Protocol

Female BALB/c mice were obtained from Taconic Biosciences. Mice were allocated to three groups (*n* = 5) as follows: (I) 200 µL IP immunization with purified bare vesicles; (II) 200 µL IP immunization with F62 vesicle vaccine (26 µg/mouse); (III) 200 µL IP immunization with F62 whole cell (26 µg/mouse). Pre-immunization bleeding was performed through the sub-mandibular blood collection method. All mice were boosted 3 weeks after initial immunization with the same vaccine formulation, and terminal bleeds were collected via cardiac puncture 5 weeks post initial immunization. All blood samples were processed using BD Microtainer serum separator tubes. Animal immunizations were performed as approved by the UMD Institutional Animal Care and Use Committee in accordance with all federal regulations and guidelines (board reference number R-JUN-18-30, approved on 30 June 2018).

### 2.14. Analysis of Elicited Antibody

*N. gonorrhoeae* strain F62ΔLgtD was grown in GCP broth and diluted to 10^8^ CFU/mL in PBS. Aliquots (50 µL) were added to wells in a 96-well plate (Thermo Fisher, Munich, Germany) and allowed to air dry overnight at room temperature. Wells were blocked for 1 h at RT with 5% skim milk in PBS, containing 0.05% Tween20 (PBST). Wells were washed three times with PBST. Various dilutions of mouse sera were added to each well (75 µL of mouse sera in blocking solution). For all test groups, pooled sera from individual mice were used in triplicates. After 24 h incubation at 4 °C, the sera was removed, and the plate washed three times with PBST. Goat α Mouse IgG +I gM HRP from Sigma Aldrich (Sigma, St. Louis, MO USA) was used as a secondary antibody at a dilution of 1:5000 (100 µL/well) and incubated for 1 h at RT. The wells were washed six times with PBST. TMB (125 µL) was added to wells, avoiding direct exposure to light and the color change monitored and absorbance read at 655 nm using automated plate reader.

## 3. Results

Despite the success of liposomal formulations of cell surface components for both in vitro and in vivo applications [17,18,19], they have significant limitations. First, cell-surface components typically reside in the hydrophobic bilayer and the solubilization and purification of these substances from the cell membrane often results in denaturation of the protein [20]. Second, since most liposomes are thermodynamically stable as films rather than as a vesicle [21], production of the liposome requires significant manipulation for formation, usually by sonication or repeated passage through a membrane to produce the unstable vesicle morphology (this mechanical stress may also denature the protein(s) incorporated into the leaflet). Third, liposomal preparations prepared by sonication or membrane extrusion are heterogeneous in size, ranging from 300 nm to 20 microns [22], and production of homogeneously sized liposomes requires some form of post-formation processing [23]. Finally, unless further modified, liposomal formulations are unstable in biological media due to pH and ionic strength issues and are thermodynamically unstable with regard to morphology, precluding long-term storage of liposome suspensions [24]. Accordingly, most liposomal preparations require either reconstitution immediately prior to use or addition of additional components to the formulation mixture to stabilize the liposome in the vesicle morphology.

### 3.1. Formation of Surfactant Vesicles (SVs)

**Scheme 1:**
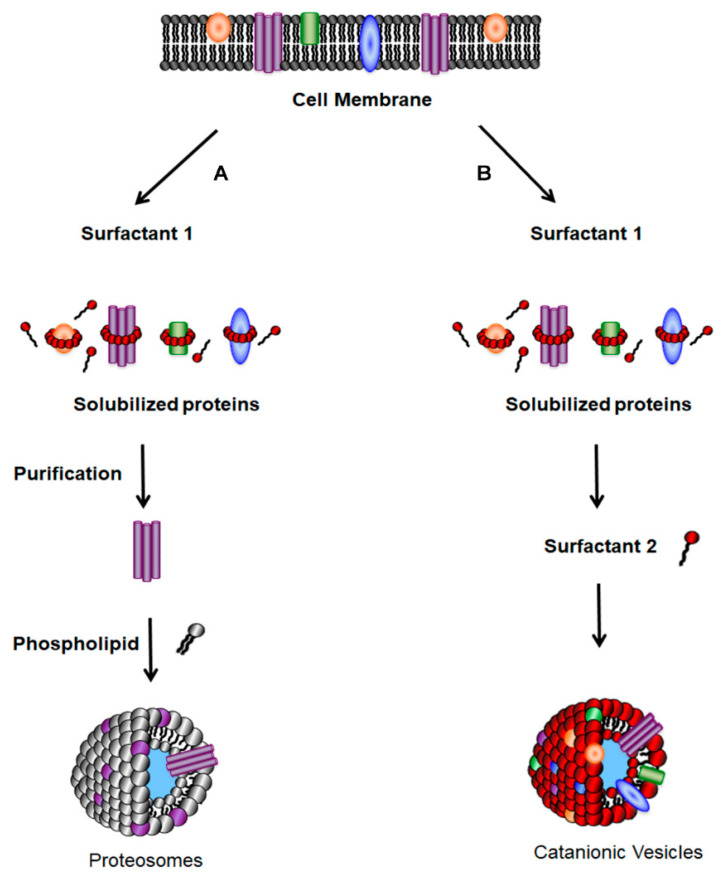
To incorporate membrane proteins into vesicles, bacteria are grown in broth and collected by centrifugation. The left-hand column (labeled A) represents how membrane proteins are incorporated into liposomes. The protein(s) of interest are released from the membrane by the addition of a surfactant, are purified in some manner, and then mixed with phospholipids. There are converted into proteasomes using one of many liposome formulation protocols. Surfactant vesicles are prepared by extracting the membrane with potentially the same surfactant (SDBS). Proteins can be directly incorporated into vesicles by adding a second surfactant (CTAT), with simple mixing. Unincorporated surfactant can be removed by size exclusion chromatography.

There are a number of strategies employed for the insertion of proteins into liposomes, including mechanical means, freeze–thaw methods, organic solventmediated reconstitution, direct incorporation into preformed liposomes, or detergent-mediated reconstitution [4,25]. Detergent-mediated reconstitution is the most commonly used method, because detergents are often involved during the initial solubilization step for the isolation of cellular membrane proteins. As outlined in Scheme 1 (left hand column), the detergent-mediated reconstitution procedure developed by Rigaud begins with the addition of detergent to the native membrane to solubilize proteins in a lipid–detergent solution [4,25]. After protein purification, excess detergent is removed by dialysis, gel chromatography, or absorption onto hydrophobic resins to allow for the formation of closed lipid vesicles [26]. Due to their lipophilic nature, membrane proteins have a predilection to embed themselves within the liposomal bilayer to form proteoliposomes [27]. While preparation of SVs uses the same initiation step, their formation occurs spontaneously by the addition of the second surfactant (Scheme 1, right hand column).

### 3.2. Stability of SVs

SVs, initially reported by Kaler and coworkers [28], have numerous advantages over their liposomal counterparts as a vehicle for extraction and presentation of cell surface components. To demonstrate their thermodynamic stability, SVs were formulated and monitored by dynamic light scattering over an extended period. As shown in Table 1, SVs that formed after 1 h were comparable in size and opacity to vesicle samples stored for >6 months. This result indicates that SVs should be stable indefinitely at room temperature.

### 3.3. Comparative Properties of SVs and Liposomes

The ability to create complex nanomaterials teamed with the robust character of SVs, particularly in biological buffers [29], would make the resulting functionalized vesicles attractive for vaccine formulations and various host–cell interaction studies. The properties of these functionalized vesicles and their comparison to liposomes are described in Table 2. Most notably, SVs are formed from inexpensive, commercially available surfactants, they form spontaneously with only mixing involved. The resulting SVs are readily purified by size exclusion chromatography (SEC) and, once purified, possess long-term and enhanced stability when compared to their liposomal counterparts [28].

### 3.4. Extraction of N. gonorrhoeae Components by SVs

**Figure 1:**
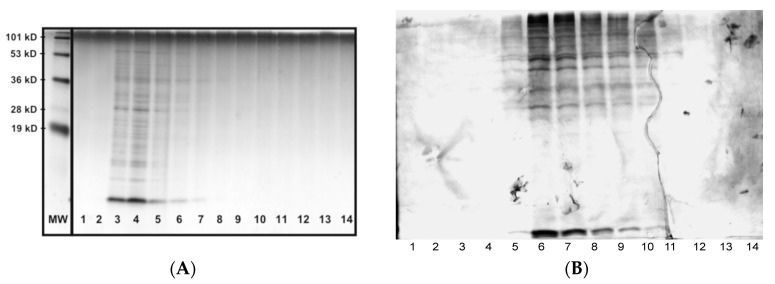
Gel filtration of SV extracts and whole cell lysates. Panel (**A**) represents the elution profile (1 mL fractions, 10 mL Sephadex 100 column) of SVs, and Panel (**B**) represents the elution profile (1 mL fractions, 10 mL Sephadex 100 column) of a whole cell lysate. Lanes were loaded with 1 μL of sample and analyzed by SDS–PAGE on 16.5% Tris–Tricine gels, followed by silver staining. Lanes 1 and 2 represent the void volume. MW represents the approximate location of various molecular weight marker bands.

To demonstrate the utility of SVs in extracting surface components, SVs extractions of *N. gonorrhoeae* were prepared by a variety of methods as described in the Materials and Methods section. The data in Figure 1 are representative of the purification data obtained when the various surfactant preparations were purified. The data in Panel A indicate that surfactant particles containing cellular components are seen primarily in fractions 3–6. This is contrasted with the data in Panel B that demonstrate that the bulk of the cellular components from the lysate are either retained on the column or elute in fractions 6–11.

After purification by gel filtration, the resulting SV extracts were tested for the presence of carbohydrate and protein using colorimetric assays [6,10]. The results of various preparation protocols are summarized in Table 3. While the total amount of protein and carbohydrate varied with the extraction protocol, each protocol was able to incorporate significant levels of bacteria-derived proteins and carbohydrates into the SVs. While the carbohydrate and protein assays quantitatively determine the amount of carbohydrate and protein contained within the vesicles, they lack the ability to determine the specific gonococcal components present in the SVs extracts.

**Figure 2:**
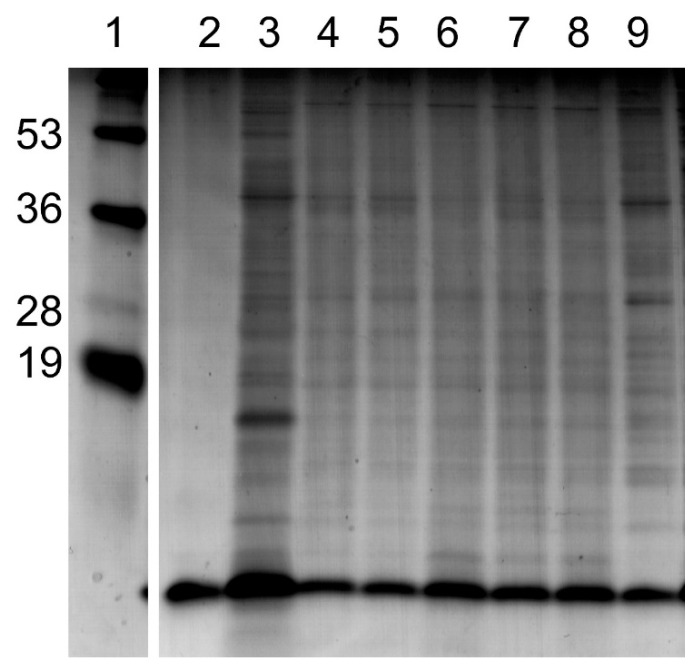
SDS-PAGE analysis of SV preparations. The lanes represent: 1, molecular weight standard (numbers on left in kD); 2, purified LOS isolated from F62ΔlgtD; 3, whole cell lysate pellet of *N. gonorrhoeae*; 4, corresponded to SV fractions prepared from extraction methods 1; 5, corresponded to SV fractions prepared from extraction methods 2; 6, corresponded to SV fractions prepared from extraction methods 3; 7, corresponded to SV fractions prepared from extraction methods 4; 8, corresponded to SV fractions prepared from extraction methods 5; 9, purified cell lysate disrupted by SDBS and purified by gel filtration.

In order to demonstrate that only a subset of the cellular components of the bacteria were incorporated into the vesicles, the SVs extraction samples were subjected to electrophoresis then compared to the whole cell lysate and original cell pellet of *N. gonorrhoeae*; the results of the analysis are shown in Figure 2. The presence of lipooligosaccharide (LOS) from *N. gonorrhoeae* in vesicles was suggested by the presence of bands with electrophoretic mobility similar to that of purified F62ΔlgtD LOS. We had previously demonstrated (unpublished results) that bacterial LOS is readily incorporated into SVs because the lipid A portion of LOS should be solubilized by the extraction and then deposited into the lipophilic bilayer of the SVs.

### 3.5. Composition of Components Incorporated into SVs

**Figure 3:**
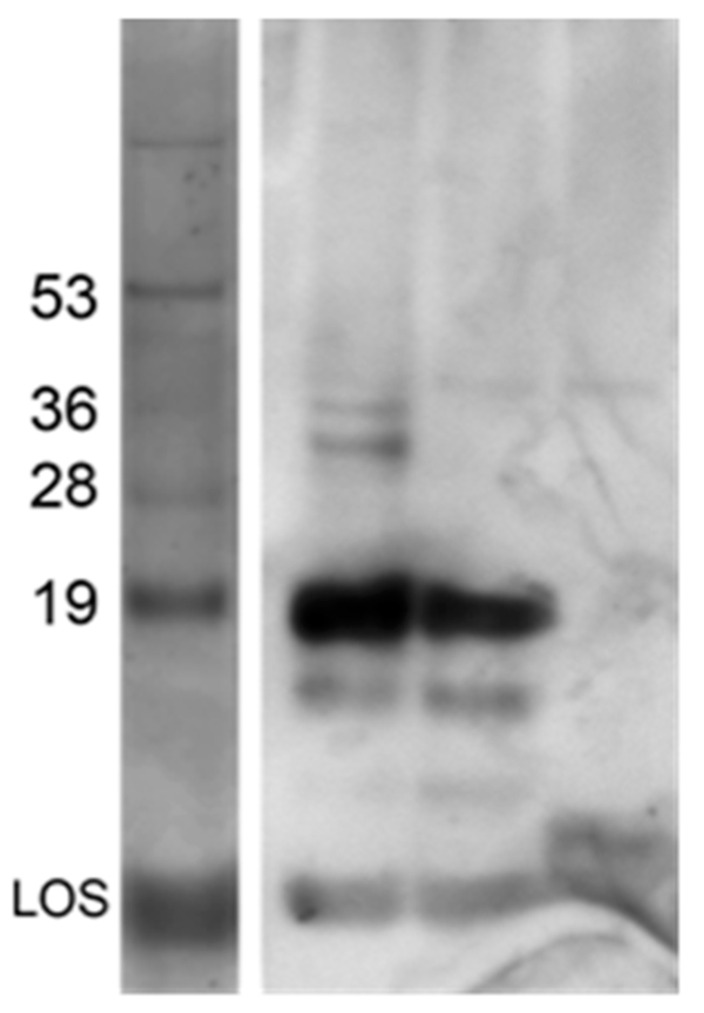
Vesicles contain immunogenic proteins. Samples were analyzed after electrophoresis through a 16.5% Tris–Tricine gel. Western blotting of vesicle-containing fractions (lane 1), lysate (lane 2), and culture supernatant (lane 3). Protein standards were spiked with LOS isolated from F62ΔlgtDfix. Components in the Western blot were detected using a polyclonal sera raised against whole cell GC.

As an initial measure of what was incorporated into SVs, we compared the reactivity of extracted components in SVs as detected by Western blotting to those contained in a whole cell extract. Analysis of this blot showed the presence of LOS and the two predominant membrane proteins pilin (18 kD) and Opa (25–30 kD) (Figure 3).

Mass spectrometry analysis was performed on SVs containing gonococcal cell extracts and 157 gonococcal proteins were identified in the extracts. The identity of each protein and the list of peptides identified are presented in Supplemental Tables S1 and S2. This bioinformatic analysis identified many of the known outer membrane proteins and membrane associated proteins found in the *N. gonorrhoeae*. For example, this analysis identified the major outer membrane protein porin P.IB, the outer membrane protein PIII, the major lipoprotein, components of the antibiotic resistance efflux pump, pilin and proteins associated with pilin assembly, numerous transport proteins, and putative two-component transport system proteins. The mass spectral analysis also identified numerous ribosomal proteins and proteins involved in energy generation. These latter proteins, while not components of the outer membrane, represent major proteins found in the bacterium and would be expected to contaminate outer membrane extracts assuming that there was also partial disruption of the bacterial membrane.

### 3.6. Surface Expression of Incorporated Components

**Figure 4:**
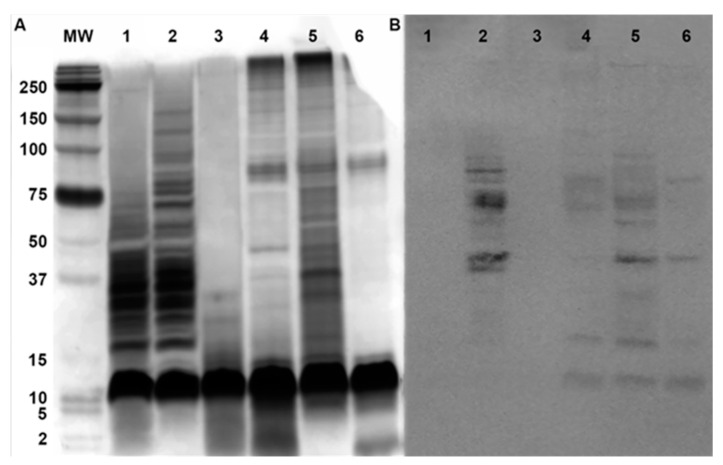
Susceptibility of proteins to enzymatic digestion. The panels represent: (**A**) SDS–PAGE and (**B**) Western Blot of duplicate gel. The lanes represent: MW, molecular weight standard (numbers on left in kD); 1, whole cell lysate (WCE) treated with trypsin for 24 h; 2, WCE untreated; 3, WCE treated with proteinase K for 24 h; 4, SVs treated with trypsin for 24 h; 5, SVs untreated; 6, SVs treated with proteinase K.

As a measure of demonstration of surface expression of extracts formulated in vesicles, purified vesicles were subjected to enzymatic digestion. Proteinase K protection experiments were performed to determine if bacterial proteins are localized to the interior of the vesicles. Loaded vesicles were digested with proteinase K to determine if being associated with vesicles protected proteins. Both vesicle samples and the whole cell lysate showed complete digestion using this enzyme (Figure 4). Since proteinase K is a nonspecific digestion enzyme, these results demonstrate that any protein at the surface of vesicles was digested by the enzyme. This proteolytic enzyme does not digest LOS.

Digestion of vesicles using trypsin gave a different pattern. Digested proteins in the form of peptide units were seen in great concentrations at the bottom of the gel (Figure 4). The vesicle samples showed protection for a few proteins, while the whole cell lysate fraction was completely cleaved by trypsin. The most logical interpretation of these digestion experiments is that SVs contain proteins embedded in the leaflet bilayer, where the portion of the protein embedded into the leaflet is protected from cleavage from trypsin, but not proteinase K.

### 3.7. Cytotoxicity of SVs

**Figure 5:**
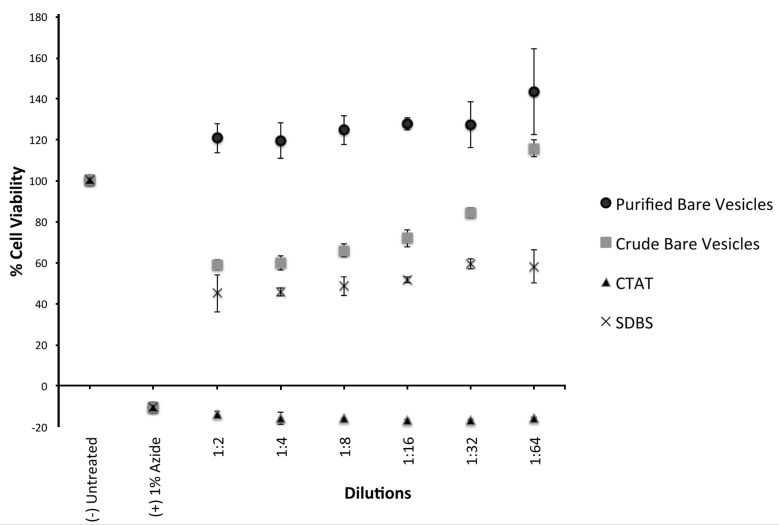
Cytotoxicity of CVs and components. Aliquots of vesicles or their components were incubated with THP-1 cells for 24 h and the viability assessed using an Alamar Blue assay from Biorad. 1% sodium azide was used as a killing control. The data were normalized to 100% of the reading obtained in the assay with untreated cells. All data points were assayed in triplicate. Error bars represent one standard deviation.

To determine if SVs were toxic to human cells, we assessed the toxicity of CTAT, SDBS, and purified vesicles on THP-1 cells, a cell line used as a model for human monocytes [30]. Sodium azide (1% final concentration) was used as a positive killing control. After 24 h incubation with various concentrations of surfactants and surfactant components, viability was measured using the Alamar blue assay from Biorad. All assays were done in triplicate, and the absorbance normalized to the untreated well. The concentration of the stock solutions of CTAT and SDBS were normalized to 13.4 mM SDBS or 5.38 mM CTAT, as these are the concentrations of the components in purified vesicles. The results are illustrated in Figure 5 shows cytotoxicity of purified bare vesicles and crude (unpurified) bare vesicles, as well as the vesicle constituents. The data indicate that CTAT is highly toxic and SDBS has intermediate toxicity. However, when CTAT and SDBS were formulated as vesicles, and the unincorporated components were purified away by column chromatography, they were not toxic. In the absence of purification, the resulting vesicle preparations were toxic, most likely reflecting the presence of a small amount of unincorporated CTAT present in the solution. From these data, we concluded that incorporation of CTAT and SDBS into vesicles eliminated their toxicity. However, these data demonstrates that in the absence of purification, the vesicles contain unincorporated components, and that the toxicity of these components would preclude using vesicles for biological applications.

### 3.8. Cytokine Induction by SVs

**Figure 6:**
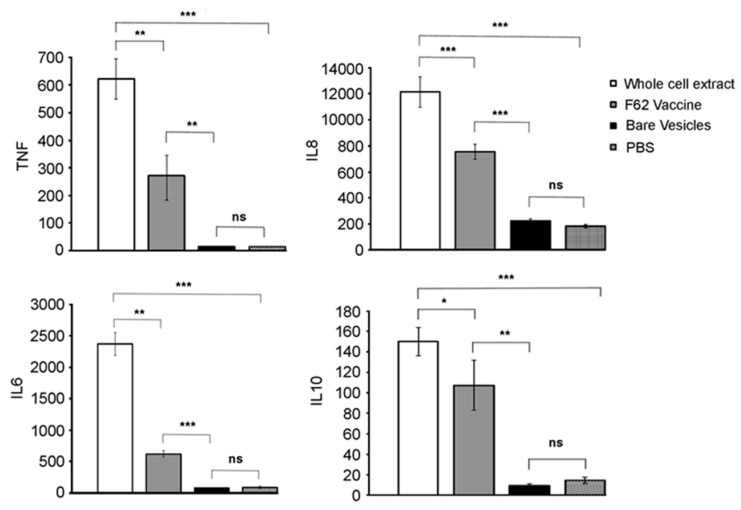
Cytokine induction by SVs, and SVs formulated with *N. gonorrhoeae*. Human THP-1 cells, grown in RPMI-1640 supplemented with 10% FBS (no antibiotics) were incubated at 37 °C for 24 h with the indicated extracts, broth from each well collected and cytokine expression determined using Invitrogen Human Cytokine Elisa Kits. All assays were performed in triplicate. Statistical significance was determined using a student *t*-test. *, *p* < 0.05, **, *p* < 0.01, ***, *p* < 0.001. ns is not significant. Error bars represent one standard deviation.

When macrophages are activated they secrete cytokines, such as tumor necrosis factor (TNF), IL-6, and IL-8 [31]; IL-10 is an anti-inflammatory cytokine [32]. In order to assess the immune potentiating properties of SVs, we incubated THP-1 cells with SVs and assayed for the expression of these cytokines by ELISA. The data (Figure 6) indicate that when SVs were incubated with THP-1 cells, they did not induce production of these cytokines, indicating that bare vesicles are not immunostimulatory.

One potential use of SVs is as a vaccine delivery system. We used SVs formulated to contain *N. gonorrhoeae* surface components, as described above. WCEs of gonococci were prepared and diluted such that the protein level contained in the extracts was equivalent to the protein level contained in the SVs, as measured by the BCA assay. Equal amounts of protein (from WCE or contained within SVs) were added to THP-1 cells, and the level of cytokine expression measured. As expected, the WCE was significantly immunostimulatory, when compared to unstimulated controls. SVs formulated with *N. gonorrhoeae* components, while immunostimulatory, were significantly less immunostimulatory than WCEs. These data suggest that incorporation of surface components into SVs reduced the stimulatory potential of the gonococcal components.

### 3.9. Immunogenicity of GC-Formulated SVs

**Figure 7:**
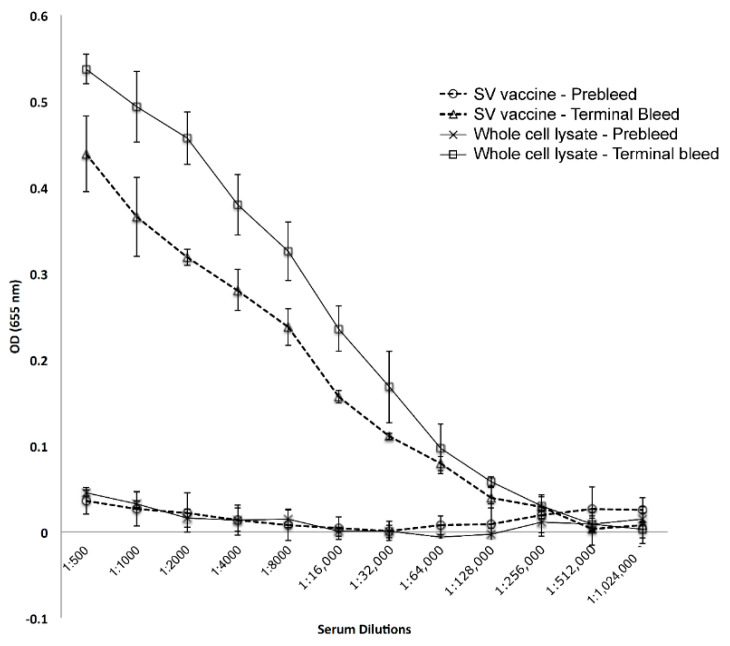
Immunogenicity of SV vaccine and whole cell extracts. BalbC mice were immunized with SVs or whole cell extracts, where the immunizing doses were normalized to 26 µg of protein per immunization. Mice were boosted at three weeks, and terminal blood draws were collected after two addition weeks. Elicited antibody levels were determined by ELISA (total IgG and IgM).

In order to assess the immunopotentiating activity of GC antigens incorporated into SVs, Balb-C mice were immunized with equal amounts of protein (formulated or not), and the level of antibody induced measured after one boost. High levels of antibody were induced by both preparations (Figure 7). Since the level of antibody induced by the two preparations were similar, it suggests that SVs can serve as a vehicle for vaccine formulation. Future studies will determine the breadth of the antigens capable of inducing antibody, and the functionality of the antibody induced.

## 4. Discussion

Several approaches have been developed for antigen display for vaccine formulation (virus-like nanoparticles, liposome-based antigen delivery systems, niosomes etc. These platforms require preparation of inert virus backbones or distinct lipid formulations (see Vijayan et al. for recent review [33]). SVs possess a number of similarities to these systems but have distinct advantages (see Table 2). SVs are distinguished from liposomes and other vesicle forming systems in that they form spontaneously upon mixing of oppositely charged single-tailed surfactants. Because SVs are formed from inexpensive, commercially available common surfactants and the vesicles form spontaneously with only mixing involved, these properties make them attractive due to ease of handling, preparation, and storage. We have previously shown by cryo-electron microscopy that the vesicles are relatively homogeneous in structure with a diameter of 75 ± 20 nm [7]. Their stability at room temperature, ease of formulation, and inexpensive components further highlight some of their advantages. From a practical standpoint, individually, the components have been approved for human use by the US FDA.

As a result of their unique biophysical properties, SVs formulated in the presence of bacteria can be easily purified from unincorporated components by simple size exclusion column chromatography (Figure 1). Depending on the formulation method employed, one can prepare SVs differing in overall composition (Figure 2), providing opportunities for studying how different components interact in biological systems. We show that proteins incorporated in SVs are surface exposed, because they are susceptible to enzymatic digestion (Figure 4) and are able to elicit a strong immune response. For example, treatment of SVs derived from the extraction of *N. gonorrhoeae* incorporates proteins formerly embedded into the bacterial membrane; however, upon treatment of these bacteria-derived SVs with proteinase K or trypsin, the proteins are readily digested.

If SVs are to be deployed in biological systems for vaccine formulation or drug delivery, they must be biocompatible. The data in Figure 5 show that while the individual surfactants are quite toxic, at the highest concentration that they can be formulated, purified SVs exhibit no toxicity using THP-1 cells as a readout. The high stability of SVs in biological media indicates that the individual surfactants are not shed into the medium (buffer or serum). The need for purification of SVs by size exclusion chromatography is to remove trace amounts of unincorporated surfactants. These surfactant micellular structures exhibited significant toxicity. We also show that purified SVs are not immunostimulatory, as they failed to induce any of the cytokines assayed (Figure 6). Because SVs, when formulated to contain gonococcal components, were highly immunostimulatory (Figure 7), it suggests that SVs can form the basis of a new vesicular antigen presentation system.

## 5. Conclusions

SVs are soft nanomaterials that can incorporate antigens, making them ideal vehicles for vaccine formulation and membrane protein study. The ability to directly extract membrane components from pathogenic bacteria and a mass spectral method to determine the peptides that have been incorporated into the SVs leaflet demonstrate their significant superiority to conventional liposomes. The vesicle extraction protocols introduced offer potential enrichment of LOS and other membrane components from *N. gonorrhoeae*. The resulting functionalized SVs are stable at room temperature for prolonged periods of time. Bioinformatic analysis of the peptide components in the SVs demonstrated that the extraction protocols selectively removed cell surface components from the bacterial membrane. The ability to reduce the toxicity of WCEs while retaining immunogenicity suggests that SVs have the potential to be superior to liposomes in a wide variety of biological applications.

## Figures and Tables

**Table 1:** Temporal stability of vesicles by dynamic light scattering (DLS).

Time	Hydrodynamic Radius (nm) (± <2 nm) *
1 h	72
1 week	75
1 month	74
5 months	78
8.5 months	75

A stock solution of SVs was analyzed to determine the shelf life of solutions stirred at room temperature. * The standard deviation of all vesicle suspensions were <2 nm.

**Table 2:** Comparison of physicochemical features of Surfactant Vesicles (SVs) to liposomes.

	Catanionic Vesicles	Phospholipid Liposomes
Constituents	Inexpensive surfactants ($1/kg)	Expensive phospholipids ($1000/kg)
Formation	Spontaneous	Sonication, extrusion, electroformation, and biphasic evaporation techniques
Stability	Years at room temp. in saline	Days at room temp. in saline
Entrap ionic solutes or nonpolar molecules	Highly efficient ^1^	Inefficient
pH	2–12 ^1^	6–8
Heating	85 °C max ^1^	42 °C max
Sterilization	Maintain integrity after autoclaving ^1^	Integrity disrupted by autoclaving

^1^ Data supporting these conclusions are found in Stocker PhD thesis, University of Maryland [29].

**Table 3:** Total protein and carbohydrate concentrations in vesicle extract samples determined by colorimetric BCA and carbohydrate assays. The five cationic extraction protocols yielded different quantities of protein and carbohydrate. The protein concentration of the anionic vesicles was not tested (NT).

Protocol	Protein (μg/mL)	Carbohydrate (μg/mL)	Protein/Carbohydrate
1	333	38	8.76
2	309	62	4.98
3	408	56	7.29
4	346	44	7.86
5	341	26	13.12

## Supplementary Materials

The following are available online at https://www.mdpi.com/1999-4923/12/9/787/s1, Table S1: Proteins identified in vesicles, Table S2: Peptides identified by mass spectrometry.

## References

[1] Scott J.D., Pawson T. (2009). Cell signaling in space and time: Where proteins come together and when they’re apart. Science.

[2] Boyle E.C., Finlay B.B. (2003). Bacterial pathogenesis: Exploiting cellular adherence. Curr. Opin. Cell Biol..

[3] Lazar V., Chifiriuc M.C. (2010). Architecture and physiology of microbial biofilms. Roum. Arch. Microbiol. Immunol..

[4] Rigaud J.L. (2002). Membrane proteins: Functional and structural studies using reconstituted proteoliposomes and 2-D crystals. Braz. J. Med. Biol. Res..

[5] Akbarzadeh A., Rezaei-Sadabady R., Davaran S., Joo S.W., Zarghami N., Hanifehpour Y., Samiei M., Kouhi M., Nejati-Koshki K. (2013). Liposome: Classification, preparation, and applications. Nanoscale Res. Lett..

[6] Park J., Rader L.H., Thomas G.B., Danoff E.J., English D.S., DeShong P. (2008). Carbohydrate-Functionalized Catanionic Surfactant Vesicles: Preparation and Lectin Binding Studies. Soft Matter.

[7] Thomas G.B., Rader L.H., Park J., Abezgauz L., Danino D., DeShong P., English D.S. (2009). Carbohydrate Modified Catanionic Vesicles: Probing Multivalent Binding at the Bilayer Interface. J. Am. Chem. Soc..

[8] Song W., Ma L., Chen W., Stein D.C. (2000). Role of lipooligosaccharide in *opa*-independent invasion of *Neisseria gonorrhoeae* into human epithelial cells. J. Exp. Med..

[9] Stein D.C., Petricoin E.F., Griffiss J.M., Schneider H. (1988). Use of transformation to construct *Neisseria gonorrhoeae* strains with altered lipooligosaccharides. Infect. Immun..

[10] Smith P.K., Krohn R.I., Hermanson G.T., Mallia A.K., Gartner F.H., Provenzano M.D., Fujimoto E.K., Goeke N.M., Olson B.J., Klenk D.C. (1985). Measurement of protein using bicinchoninic acid. Anal. Biochem..

[11] Chevallet M., Luche S., Rabilloud T. (2006). Silver staining of proteins in polyacrylamide gels. Nat. Protoc..

[12] Towbin H., Staehelin T., Gordon J. (1979). Electrophoretic transfer of proteins from polyacrylamide gels to nitrocellulose sheets: Procedure and some applications. Proc. Natl. Acad. Sci. USA.

[13] LeVan A., Zimmerman L.I., Mahle A.C., Swanson K.V., DeShong P., Park J., Edwards V.L., Song W., Stein D.C. (2012). Construction and characterization of a derivative of *Neisseria gonorrhoeae* strain MS11 devoid of all *opa* genes. J. Bacteriol..

[14] Sandlin R.C., Stein D.C. (1994). Role of phosphoglucomutase in lipooligosaccharide biosynthesis in *Neisseria gonorrhoeae*. J. Bacteriol..

[15] Shevchenko A., Tomas H., Havlis J., Olsen J.V., Mann M. (2006). In-gel digestion for mass spectrometric characterization of proteins and proteomes. Nat. Protoc..

[16] Choksawangkarn W., Kim S.K., Cannon J.R., Edwards N.J., Lee S.B., Fenselau C. (2013). Enrichment of plasma membrane proteins using nanoparticle pellicles: Comparison between silica and higher density nanoparticles. J. Proteome Res..

[17] Jesorka A., Orwar O. (2008). Liposomes: Technologies and Analytical Applications. Ann. Rev. Anal. Chem..

[18] Chang H.I., Yeh M.K. (2012). Clinical development of liposome-based drugs: Formulation, characterization, and therapeutic efficacy. Int. J. Nanomed..

[19] Schwendener R.A. (2014). Liposomes as vaccine delivery systems: A review of the recent advances. Ther. Adv. Vaccines.

[20] Hjerten S., Sparrman M., Liao J. (1988). Purification of membrane proteins in SDS and subsequent renaturation. Biochim. Biophys. Acta.

[21] Lasic D.D. (1990). On the thermodynamic stability of liposomes. J. Colloid Interface Sci..

[22] Johnson S.M., Bangham A.D. (1969). Potassium permeability of single compartment liposomes with and without valinomycin. Biochim. Biophys. Acta.

[23] Bozzuto G., Molinari A. (2015). Liposomes as nanomedical devices. Int. J. Nanomed..

[24] Alipour E., Halverson D., McWhirter S., Walker G.C. (2017). Phospholipid Bilayers: Stability and Encapsulation of Nanoparticles. Annu. Rev. Phys. Chem..

[25] Rigaud J.-L., Pitard B., Philippot J., Schuber F. (1994). Liposomes as Tools for the Reconstitution of Biological Systems. Liposomes as Tools in Basic Research and Industry.

[26] Girard P., Pécréaux J., Lenoir G., Falson P., Rigaud J.L., Bassereau P. (2004). A new method for the reconstitution of membrane proteins into giant unilamellar vesicles. Biophys. J..

[27] Shen H.H., Lithgow T., Martin L. (2013). Reconstitution of membrane proteins into model membranes: Seeking better ways to retain protein activities. Int. J. Mol. Sci..

[28] Kaler E.W., Murthy A.K., Rodriguez B.E., Zasadzinski J.A. (1989). Spontaneous vesicle formation in aqueous mixtures of single-tailed surfactants. Science (New York, NY ).

[29] Stocker L. (2013). Catanionic SUrfactant Vesicles: Technology for Vaccine Depvelopment and Targetted Drug Delivery Applications. Ph.D. Thesis.

[30] Bosshart H., Heinzelmann M. (2016). THP-1 cells as a model for human monocytes. Ann. Transl. Med..

[31] Arango Duque G., Descoteaux A. (2014). Macrophage cytokines: Involvement in immunity and infectious diseases. Front. Immunol..

[32] Couper K.N., Blount D.G., Riley E.M. (2008). IL-10: The master regulator of immunity to infection. J. Immunol..

[33] Vijayan V., Mohapatra A., Uthaman S., Park I.K. (2019). Recent Advances in Nanovaccines Using Biomimetic Immunomodulatory Materials. Pharmaceutics.

