# Supplementary material for: Extraction of Membrane Components from Neisseria gonorrhoeae Using Catanionic Surfactant Vesicles: A New Approach for the Study of Bacterial Surface Molecules
Source: Pharmaceutics. 2020 Aug 20;12(9):787. doi: 10.3390/pharmaceutics12090787 (PMC7559012; doi:10.3390/pharmaceutics12090787)
Supplement: Supplementary file 1 [file pharmaceutics-12-00787-s001.pdf]

pharmaceutics

Article

Supplementary Materials: Extraction of Membrane Components from *Neisseria gonorrhoeae* Using Catanionic Surfactant Vesicles: A New Approach for the Study of Bacterial Surface Molecules

Daniel C. Stein, Lenea H. Stocker, Abigail E. Powell, Salsawi Kebede, David Watts, Emma Williams, Nicholas Soto, Avantika Dhabaria, Catherine Fenselau, Shweta Ganapati and Philip DeShong

Table S1. Proteins identified in vesicles.

UniProtAC	Description	# of spectra identified
Q5F5V7	Major outer membrane protein porin P.IB	270
Q5F939	Pyruvate dehydrogenase E1 component	154
Q5F865	Putative peroxiredoxin family protein/glutaredoxin	153
Q5F9Q2	Cysteine synthase	116
Q5F9M1	Putative ABC transporter, periplasmic binding protein, amino acid	109
Q5F6I1	Outer membrane protein PIII	83
Q5F7E7	Aconitate hydratase 2	52
Q5F5P4	Putative uncharacterized protein	49
Q5F905	30S ribosomal protein S1	45
Q5F7F3	Peptidyl-prolyl cis-trans isomerase	36
Q5F942	Putative dihydrolipoamide dehydrogenase	34
Q5F501	Lipoprotein	33
Q5F724	Antibiotic resistance efflux pump component	33
Q5F5Y3	Putative ribonuclease E	32
Q5F6M3	Cell division protein FtsZ	32
Q5F725	Antibiotic resistance efflux pump component	32
Q5F7W0	Putative uncharacterized protein	31
Q5F7T8	Putative isocitrate dehydrogenase	27
Q5F5J3	Putative glyceraldehyde 3-phosphate dehydrogenase C	26
Q5F940	Putative dihydrolipoamide acetyltransferase component of pyruvate dehydrogenase complex	26
Q5FA20	Putative phosphotransacetylase	26
Q5F874	Putative 2-oxoglutarate dehydrogenase, E1 component	25
Q5F4Y0	Putative 3-oxoacyl-[acyl-carrier protein] reductase	22
Q5F824	Acetate kinase	22
Q5F5L5	Twitching motility/pilus retraction protein	21
Q5F649	Putative thiol:disulphide interchange protein	21
Q5F731	Glutamate dehydrogenase	21
Q5F809	Lipid modified azurin protein	21
Q5F7W9	Putative ClpB protein	20
Q5F601	Catalase	19
Q5FAI4	Putative fructose-bisphosphate aldolase	19
Q5F6N6	Outer membrane opacity protein D	18
Q5F7Y3	Putative transketolase	18
Q5F8H0	Putative GTP-binding protein	18
Q5F6Q5	Putative ABC transporter, periplasmic binding protein, polyamine	17
Q5F7L8	ABC transporter, periplasmic histidine-binding protein	17
Q5FA34	Phosphoenolpyruvate synthase	17
Q5F6R0	Putative aspartyl-tRNA synthetase	16

Q5F6V3	Putative alcohol dehydrogenase	14
Q5F726	Multidrug efflux pump channel protein	14
Q5F8I5	Genome-derived Neisseria antigen 1220	14
Q5F716	Putative cytochrome c oxidase subunit	13
Q5F759	Putative cytochrome	13
Q5F7X2	Putative uncharacterized protein	13
Q5F875	Putative dihydrolipoamide succinyltransferase E2 component	13
Q5F879	Succinyl-CoA ligase [ADP-forming] subunit alpha	12
Q5FA46	Putative zinc-binding alcohol dehydrogenas	12
Q5FAH2	Carbamoyl-phosphate synthase large chain	11
Q5F598	Putative cytochrome C1	10
Q5F8H4	Inosine-5'-monophosphate dehydrogenase	10
Q5FAB8	Putative uncharacterized protein	10
Q5FAG7	Pilus-associated protein	10
Q5F5N5	Pyruvate kinase	9
Q5F5W8	Outer membrane protein assembly factor BamA	9
Q5F6F9	Glutamine synthetase	9
Q5F6M2	Cell division protein ftsA	9
Q5F9Z4	Putative malic enzyme, malate dehydrogenase/oxaloacetate-decarboxylating	9
Q5F666	Signal recognition particle protein	8
Q5F8Q3	Putative phosphogluconate dehydratase	8
Q5F9F5	Ribose-phosphate pyrophosphokinase	8
Q5F9Z8	Cell division protein ZipA	8
Q5FAC8	Putative pilus assembly protein	8
Q5F5I9	Putative electron transfer flavoprotein beta-subunit	7
Q5F798	Putative N utilisation substance protein A	7
Q5F898	Putative D-lactate dehydrogenase	7
Q5F8T2	Putative uncharacterized protein	7
Q5FAD0	Pilus assembly protein	7
Q5F4Z9	Putative chromosome segregation protein	6
Q5F693	Pilus assembly protein	6
Q5F7T9	Alpha-2,3-sialyltransferase	6
Q5F842	Aspartokinase	6
Q5F873	Citrate synthase	6
Q5F8J6	Putative DNA-binding protein Hu	6
Q5F9K8	Delta-aminolevulinic acid dehydratase	6
Q5FA28	Putative ABC transporter, periplasmic binding protein, polyamine	6
Q5FA72	Putative aminopeptidase N	6
Q5FA80	Putative ATP-dependent RNA helicase	6
Q5F697	Putative branched-chain amino acid aminotranferase	5
Q5F6A4	Putative uncharacterized protein	5
Q5F6U0	Putative aminopeptidase	5
Q5F7F1	Probable cytosol aminopeptidase	5
Q5F7Y0	Single-stranded DNA-binding protein	5
Q5F848	Putative uncharacterized protein	5
Q5F876	Dihydrolipoyl dehydrogenase	5
Q5F884	Putative uncharacterized protein	5
Q5F8D7	Putative uncharacterized protein	5
Q5F8K6	Putative peptidyl-prolyl cis-trans isomerase	5
Q5F8K8	Putative ABC-transporter, ATP-binding protein	5
Q5F8X1	Putative L-lactate dehydrogenase	5
Q5F8Y0	DNA gyrase subunit A	5
Q5F933	Putative uncharacterized protein	5
Q5F9I4	Putative cold shock protein	5
Q5FAC5	Putative cytochrome	5
Q5F4X7	Malonyl CoA-acyl carrier protein transacylase	4
Q5F533	Putative DNA polymerase I	4
Q5F5M0	DNA-binding competence protein 2	4
Q5F6I5	Putative ABC transporter, ATP-binding protein	4
Q5F7C9	Putative uncharacterized protein	4
Q5F7G1	Glutathione synthetase	4
Q5F8Q4	Putative khg/kdpg 4-hydroxy-2-oxoglutarate aldolase	4
Q5F8W0	Putative ATP-dependent RNA helicase	4

Q5F8Z6	Ribonucleoside-diphosphate reductase	4
Q5F9L7	Peptidyl-prolyl cis-trans isomerase	4
Q5F9P7	Putative twitching motility-like protein	4
Q5F9S3	Putative uncharacterized protein	4
Q5F9W7	Putative ABC transporter, ATP-binding protein, amino acid	4
Q5FA35	Transcription termination factor Rho	4
Q5FA55	Putative two-component system transcriptional response regulator	4
Q5FAG0	Putative GTP-binding protein	4
Q5FAH6	Acetyl-CoA carboxylase, biotin carboxylase	4
Q5F571	Putative peptide methionine sulfoxide reductase	3
Q5F573	Putative uncharacterized protein	3
Q5F5L4	Putative twitching motility-like protein	3
Q5F668	Phospho-2-dehydro-3-deoxyheptonate aldolase	3
Q5F7E1	Putative uncharacterized protein	3
Q5F7J5	Putative nitrogen regulatory protein P-II	3
Q5F7X0	Tryptophan--tRNA ligase	3
Q5F839	Putative uncharacterized protein	3
Q5F870	Putative succinate dehydrogenase flavoprotein subunit	3
Q5F8C2	Putative uncharacterized protein	3
Q5F8Q1	Putative uncharacterized protein	3
Q5F8S1	Putative threonine synthase	3
Q5F8V3	Pseudouridine synthase	3
Q5F9Y5	Acetyl-coenzyme A carboxylase carboxyl transferase subunit beta	3
Q5FA17	ABC transporter, periplasmic binding protein, iron related	3
Q5F574	Putative ABC transporter, thiamine-binding periplasmic protein	2
Q5F591	Phosphoenolpyruvate-protein phosphotransferase	2
Q5F5D2	Aspartate-semialdehyde dehydrogenase	2
Q5F5E4	Putative uncharacterized protein	2
Q5F5E6	Methionine aminopeptidase	2
Q5F5I8	Putative electron transfer flavoprotein alpha-subunit	2
Q5F5R0	Transcription antitermination protein nusG	2
Q5F5X8	Putative parA family protein-putative ATPase	2
Q5F5Z8	Putative oligopeptidase A	2
Q5F621	Putative NADH dehydrogenase I chain G	2
Q5F655	Putative adenylosuccinate lyase	2
Q5F696	Enoyl-[acyl-carrier-protein] reductase [NADH]	2
Q5F6K2	Putative uncharacterized protein	2
Q5F6Y6	Aminomethyltransferase	2
Q5F766	Putative paraquat-inducible protein B	2
Q5F7M1	Putative O-succinylhomoserine sulfhydrolase	2
Q5F866	Putative dihydrolipoamide dehydrogenase	2
Q5F8D2	Putative cell-division protein	2
Q5F8Q2	Glucose-6-phosphate 1-dehydrogenase	2
Q5F8S3	Putative ferredoxin-NADP reductase	2
Q5F930	Carbonic anhydrase	2
Q5F950	Putative TonB-dependent receptor	2
Q5F9D9	Putative aminotransferases	2
Q5F9P8	5'-methylthioadenosine/S-adenosylhomocysteine nucleosidase	2
Q5F9Q9	Putative reductase	2
Q5F9W0	Outer membrane protein assembly factor BamD	2

Table S2. Peptides identified by Mass Spectrometry.

**UniProtAC**	**Description**	**Peptide**	**Right AA**	**E-value**
Q5F905	30S ribosomal protein S1	NAQGEIEVK	V	0.002
Q5F905	30S ribosomal protein S1	VGDFVTVTIESVENGFGETK	L	2.9E-06
Q5F905	30S ribosomal protein S1	GGLTVMISSIR	A	0.000067
Q5F905	30S ribosomal protein S1	AFLPGSLVDVRPVK	D	0.000002

Q5F905	30S ribosomal protein S1	AFLPGSLVDVRPVKDTSHFEGK	E	0.014
Q5F905	30S ribosomal protein S1	RAVLEATLGEER	K	0.00028
Q5F905	30S ribosomal protein S1	AVLEATLGEERK	A	0.000082
Q5F905	30S ribosomal protein S1	KALLENLQEGSVIK	G	8.6E-08
Q5F905	30S ribosomal protein S1	ALLENLQEGSVIK	G	0.000011
Q5F905	30S ribosomal protein S1	RVKHPSEVLEVGQEVEAK	V	0.000096
Q5F905	30S ribosomal protein S1	VKHPSEVLEVGQEVEAK	V	1.6E-08
Q5F905	30S ribosomal protein S1	QLGEDPWSGLTR	R	0.000049
Q5F905	30S ribosomal protein S1	RYPQATR	L	0.022
Q5F905	30S ribosomal protein S1	QLEGDPFGNFISVNDKGSLVK	G	0.00018
Q5F905	30S ribosomal protein S1	LKEGDEVEAVIVTVDRK	N	1.2E-07
Q5F905	30S ribosomal protein S1	EALNSVNAAANANAGTTSLGDLLK	A	6.2E-12
Q5F9P8	5'-methylthioadenosine/S-adenosylhomocysteine nucleosidase	GQVPQLPAR	F	0.036
Q5F9P8	5'-methylthioadenosine/S-adenosylhomocysteine nucleosidase	FASDGILIETAKR	A	0.00047
Q5FA17	ABC transporter, periplasmic binding protein, iron related	GDQLAGQIKEEGSR	S	0.000068
Q5FA17	ABC transporter, periplasmic binding protein, iron related	LEAPQVSATTVSEKEHATR	L	9.3E-07
Q5F7L8	ABC transporter, periplasmic histidine-binding protein	VASNAEFAPFESLDSK	G	0.000016
Q5F7L8	ABC transporter, periplasmic histidine-binding protein	GNVEGFDVDLMNAMAK	A	4.5E-08
Q5F7L8	ABC transporter, periplasmic histidine-binding protein	AGNFKIEFK	H	0.012
Q5F7L8	ABC transporter, periplasmic histidine-binding protein	QSMDFSDPYFEITQVVLVPK	G	2.9E-06
Q5F7L8	ABC transporter, periplasmic histidine-binding protein	VGVVTGHTGDFSVsK	L	0.00039
Q5F7L8	ABC transporter, periplasmic histidine-binding protein	LLGNDNPK	I	0.0019
Q5F7L8	ABC transporter, periplasmic histidine-binding protein	FENVPLIIK	E	0.0022
Q5F7L8	ABC transporter, periplasmic histidine-binding protein	ELENGGLDSVVSDSAVIANYVK	N	0.016
Q5F7L8	ABC transporter, periplasmic histidine-binding protein	GMDFVTLPDFTTEHYGIAVR	K	0.0031

Q5F824	Acetate kinase	LILVLNCGSSSLK	G	0.00003 2
Q5F824	Acetate kinase	VVSGGELYNESILVDDEVIAGIEK	C	1E-07
Q5F824	Acetate kinase	CIPLAPLHNPAHLLGLR	A	2.4E-07
Q5F824	Acetate kinase	GLPNVVVFDTSFHQTMPEVAYK	Y	0.00000 4
Q5F824	Acetate kinase	YGAHGTSYR	F	0.00011
Q5F824	Acetate kinase	FVADETAHFLGK	D	0.00012
Q5F824	Acetate kinase	FVADETAHFLGKDKK	D	0.0073
Q5F824	Acetate kinase	DTSMGLTPLEGLVMGTR	S	0.00009 5
Q5F824	Acetate kinase	TIEEEAAKGHKGAK	L	0.00008
Q5F824	Acetate kinase	FGNAGVITTADSK	A	3.7E-09
Q5F824	Acetate kinase	AVAVVIPTNEELMIAHDTAR	L	3.8E-06
Q5F9Y5	Acetyl-coenzyme A carboxylase carboxyl transferase subunit beta	CNHHNPLSAR	Q	0.024
Q5F9Y5	Acetyl-coenzyme A carboxylase carboxyl transferase subunit beta	FIGGSMGSVVGER	F	0.00021
Q5F9Y5	Acetyl-coenzyme A carboxylase carboxyl transferase subunit beta	TSAALHLLTEK	R	0.0056
Q5F7E7	Aconitate hydratase 2	AALGIPALPLNAQQTADLVELLK	N	0.00002 5
Q5F7E7	Aconitate hydratase 2	NPPAGEGEFLVELLAHR	V	8.8E-06
Q5F7E7	Aconitate hydratase 2	VKASFLAAVAEGSASSPLVSPK	Y	3.5E-08
Q5F7E7	Aconitate hydratase 2	ASFLAAVAEGSASSPLVSPK	Y	0.00006 1
Q5F7E7	Aconitate hydratase 2	AKVPEKITVTVFK	V	0.0092
Q5F7E7	Aconitate hydratase 2	DGITPDKPGEVGPIK	L	0.007
Q5F7E7	Aconitate hydratase 2	DGITPDKPGEVGPIKLLEELK	A	0.021
Q5F7E7	Aconitate hydratase 2	AKGHPVAYVGDVVGTGSSR	K	0.00001 3
Q5F7E7	Aconitate hydratase 2	GHPVAYVGDVVGTGSSR	K	1.2E-09
Q5F7E7	Aconitate hydratase 2	GHPVAYVGDVVGTGSSRK	S	0.00001 5
Q5F7E7	Aconitate hydratase 2	KSATNSVIWHTGEDIPFVPNKR	F	0.00029
Q5F7E7	Aconitate hydratase 2	SATNSVIWHTGEDIPFVPNKR	F	0.0034
Q5F7E7	Aconitate hydratase 2	IAPIFFNTQEDSGALPIEVDVSALK	M	1.8E-07
Q5F7E7	Aconitate hydratase 2	SQVLLDEVQAGGR	I	2.7E-08
Q5F7E7	Aconitate hydratase 2	ACGLPEGQGVRPGTYCEPR	M	0.013
Q5F7E7	Aconitate hydratase 2	MTTVGSQDTTGPMTR	D	0.00005 6
Q5F7E7	Aconitate hydratase 2	THKELPAFISTR	G	0.00084
Q5F7E7	Aconitate hydratase 2	GGVSLRPGDGVIHSWLNR	L	0.014
Q5F7E7	Aconitate hydratase 2	DLVNAIPLYAIK	Q	0.0011
Q5F7E7	Aconitate hydratase 2	LNKEPIIEYMK	S	0.0031
Q5F7E7	Aconitate hydratase 2	LLEGKSDIPVR	L	0.00009 9
Q5F7E7	Aconitate hydratase 2	ELSDEGHYGVLGR	A	0.00012
Q5F7E7	Aconitate hydratase 2	EGATVMSTSTR	N	2.5E-06
Q5F7E7	Aconitate hydratase 2	NTFVYLGSAELAAICSK	L	0.00075
Q5F7E7	Aconitate hydratase 2	LGKIPTVEEYQANIGIINEQGDKIYR	Y	0.0013
Q5F7E7	Aconitate hydratase 2	IPTVEEYQANIGIINEQGDKIYR	Y	1.6E-07
Q5F7T9	Alpha-2,3-sialyltransferase	NASDEHYTIFK	G	0.0012
Q5F7T9	Alpha-2,3-sialyltransferase	KMTYLPLFDASELKAGDETGGTVR	I	0.00001 2
Q5F7T9	Alpha-2,3-sialyltransferase	ILLGSPDKEMKEISEK	A	0.00018
Q5F7T9	Alpha-2,3-sialyltransferase	NFNIQYVAPHPR	Q	0.00001 7

Q5F6Y6	Aminomethyltransferase	AFFRKLIANDVAK	L	0.037
Q5F6Y6	Aminomethyltransferase	ALQTAGVQPCGLGAR	D	0.041
Q5F724	Antibiotic resistance efflux pump component	AQVGGIIQK	R	0.023
Q5F724	Antibiotic resistance efflux pump component	AQVGGIIQKR	L	0.000097
Q5F724	Antibiotic resistance efflux pump component	AGQPLYQIDSSTYEAGLESAR	A	5.2E-10
Q5F724	Antibiotic resistance efflux pump component	AQLATAQATLAK	A	0.00037
Q5F724	Antibiotic resistance efflux pump component	SAEAGVKAAQAAIK	S	0.0094
Q5F724	Antibiotic resistance efflux pump component	SAGINLNR	S	0.00099
Q5F724	Antibiotic resistance efflux pump component	SRITAPISGFIGQSK	V	0.00016
Q5F724	Antibiotic resistance efflux pump component	ITAPISGFIGQSK	V	3.9E-06
Q5F724	Antibiotic resistance efflux pump component	VSEGTLLNAGDTTVLATIR	Q	5E-08
Q5F724	Antibiotic resistance efflux pump component	QTNPMYVNVTQSASEVMK	L	1.3E-08
Q5F724	Antibiotic resistance efflux pump component	AAVSNDQNILMPGLYVR	V	2.9E-07
Q5F724	Antibiotic resistance efflux pump component	VLMDQVAADNAFIVPQQAVTR	G	0.00013
Q5F724	Antibiotic resistance efflux pump component	GAKDTVMIVNAQGGMEPR	E	1.7E-07
Q5F724	Antibiotic resistance efflux pump component	EWAPSENQAAAPQAGVQTASEAKPASEAK	-	0.00073
Q5F725	Antibiotic resistance efflux pump component	LSEVLSTLPATVQQYGVTVSK	A	0.0012
Q5F725	Antibiotic resistance efflux pump component	ANTDGSNIYLKDVAK	V	0.000014
Q5F725	Antibiotic resistance efflux pump component	TDATLAQVTQLAK	S	8.4E-07
Q5F725	Antibiotic resistance efflux pump component	TASGSDAVAVAGK	L	0.0051
Q5F725	Antibiotic resistance efflux pump component	ASGLFDPSTVR	A	0.0026
Q5F725	Antibiotic resistance efflux pump component	AGGLEDSPQLK	I	0.0019

Q5F725	Antibiotic resistance efflux pump component	AAAAAQGISFADIR	T	3E-08
Q5F725	Antibiotic resistance efflux pump component	TALASALSSSYVSDFPNQGR	L	8.5E-09
Q5F725	Antibiotic resistance efflux pump component	MQPADILNLTVPNK	S	1.1E-06
Q5F725	Antibiotic resistance efflux pump component	SGVAVPLSTIATVSWENGTEQSVR	F	0.0079
Q5F725	Antibiotic resistance efflux pump component	LSASPATGVSTGQAMAAVQK	M	6.9E-10
Q5F725	Antibiotic resistance efflux pump component	AGITGSDDKQY	-	0.012
Q5F842	Aspartokinase	YGGTSVGSPER	I	0.01
Q5F842	Aspartokinase	AAVTGIAFDKNQAR	I	0.00001
Q5FAH2	Carbamoyl-phosphate synthase large chain	TPASFEPSIDYVVTK	I	0.00063
Q5FAH2	Carbamoyl-phosphate synthase large chain	ELANPGPER	M	0.0066
Q5FAH2	Carbamoyl-phosphate synthase large chain	VLNDLGLRQPPNR	I	0.016
Q5FAH2	Carbamoyl-phosphate synthase large chain	IAHNEEEALVK	A	0.004
Q5FAH2	Carbamoyl-phosphate synthase large chain	AEEIGYPLVVRPSYVLGGR	A	0.01
Q5FAH2	Carbamoyl-phosphate synthase large chain	VPQYTTTAGGEAMSEGAK	S	5.5E-07
Q5F601	Catalase	TTSKCPVTHLTMNNGAPVADNQNSLTAGPR	G	4.6E-06
Q5F601	Catalase	CPVTHLTMNNGAPVADNQNSLTAGPR	G	0.0049
Q5F601	Catalase	GPLLTQDLWLNEK	L	0.00057
Q5F601	Catalase	EVIPERR	M	0.0025
Q5F601	Catalase	FTTVAGER	G	0.049
Q5F601	Catalase	LFNYADAQR	Y	0.00003
Q5F601	Catalase	QIPVNRPR	C	0.0053
Q5F601	Catalase	ALFNLMNDAQK	Q	0.000038
Q5F601	Catalase	QALFDNTAAAMGDAPDFIK	Y	0.000013
Q5F601	Catalase	QALFDNTAAAMGDAPDFIKYR	H	5.7E-06
Q5F601	Catalase	CDPAYGEGVAK	A	0.0019
Q5F6M2	Cell division protein ftsA	ITHVTTGIAGNHIR	S	1.2E-07
Q5F6M2	Cell division protein ftsA	VHIITGASTAVQNVQK	C	1.6E-08
Q5F6M3	Cell division protein FtsZ	MEFVYDVAESAVSPAVIK	V	5.2E-06
Q5F6M3	Cell division protein FtsZ	VIGLGGGGCNAINNMVANNVR	S	2E-07
Q5F6M3	Cell division protein FtsZ	SVEFISANTDAQSLAK	N	2.3E-07
Q5F6M3	Cell division protein FtsZ	RIQLGTNLTR	G	0.00084
Q5F6M3	Cell division protein FtsZ	IQLGTNLTR	G	0.00057
Q5F6M3	Cell division protein FtsZ	GLGAGANPDIGR	A	0.000015
Q5F6M3	Cell division protein FtsZ	GIAMMGSGYAQGIDR	A	6.8E-07

Q5F6M3	Cell division protein FtsZ	MATDQAISSPLLDDVTLDGAR	G	8.8E-08
Q5F6M3	Cell division protein FtsZ	GVLVNITTAPGCLK	M	1.9E-08
Q5F6M3	Cell division protein FtsZ	IVNQSAHPDLECK	F	0.0016
Q5F6M3	Cell division protein FtsZ	ITIIATGLKEK	G	0.00056
Q5F6M3	Cell division protein FtsZ	GAVDPTPAR	E	0.0055
Q5F6M3	Cell division protein FtsZ	GAVDPTPAREVEAVAPSKQEQSHNVEGMIR	T	0.017
Q5F6M3	Cell division protein FtsZ	EVEAVAPSKQEQSHNVEGMIR	T	0.000015
Q5F9Z8	Cell division protein ZipA	VRDQFGHSDKDALLNSK	T	1.6E-06
Q5F9Z8	Cell division protein ZipA	TSHVRDGKPSGGPVMMPKPQPAVK	K	0.00088
Q5F9Z8	Cell division protein ZipA	KPAKPQDSAMR	N	0.011
Q5F9Z8	Cell division protein ZipA	NLQEQDAVYIAK	Q	0.00012
Q5F873	Citrate synthase	SKSIKLNVPGR	A	0.0033
Q5F873	Citrate synthase	SIKLNVPGR	A	0.0022
Q5F873	Citrate synthase	AGLELPVLEASIGHDVVDIR	G	8.5E-06
Q5F873	Citrate synthase	IFILHADHEQNASTSTVR	L	1.2E-06
Q5F873	Citrate synthase	RDYVPAGER	-	0.0098
Q5F9Q2	Cysteine synthase	MKIANSITELIGNTPLVK	L	3.4E-09
Q5F9Q2	Cysteine synthase	IANSITELIGNTPLVK	L	0.0029
Q5F9Q2	Cysteine synthase	GLKAEVAVK	L	0.0059
Q5F9Q2	Cysteine synthase	TFGAELILTPAAEGMAGAIAK	A	3.7E-07
Q5F9Q2	Cysteine synthase	AQSLVDAHPDTYFMPR	Q	1E-07
Q5F9Q2	Cysteine synthase	QFDNEANPEVHR	K	0.00042
Q5F9Q2	Cysteine synthase	QFDNEANPEVHRK	T	0.00021
Q5F9Q2	Cysteine synthase	GPHPIQGIGAGFIPTVLNTK	I	1E-08
Q5F9Q2	Cysteine synthase	IYDSIAKVPNEAAFETAR	A	1.8E-06
Q5F9Q2	Cysteine synthase	VPNEAAFETAR	A	5.1E-07
Q5F9Q2	Cysteine synthase	EGILAGISSGAAVWSALQLAK	Q	5.3E-07
Q5F9Q2	Cysteine synthase	QPENEGKLIVVLLPSYGER	Y	3.1E-06
Q5F9Q2	Cysteine synthase	LIVVLLPSYGER	Y	0.000076
Q5F9K8	Delta-aminolevulinic acid dehydratase	LGIPMLALFPVVTANK	T	9.6E-06
Q5F9K8	Delta-aminolevulinic acid dehydratase	AQEAYNPEGLVPSTVR	A	0.000028
Q5F876	Dihydrolipoyl dehydrogenase	SQYDVVVIGAGPGGYVAAIR	A	3.7E-08
Q5F876	Dihydrolipoyl dehydrogenase	TNLPNVWAIGDVVR	G	4.2E-06
Q5F876	Dihydrolipoyl dehydrogenase	ASDEGVAVAER	I	2.7E-06
Q5F8Y0	DNA gyrase subunit A	IVGDVIGK	Y	0.049
Q5F8Y0	DNA gyrase subunit A	IIDFVDILSKPER	I	0.000023
Q5F8Y0	DNA gyrase subunit A	KNKGGQGSIAINTGER	N	0.0023
Q5F8Y0	DNA gyrase subunit A	LINLDEGETLVSLER	V	0.00024
Q5F5M0	DNA-binding competence protein 2	VKGIGPAVLAK	L	0.00015
Q5F5M0	DNA-binding competence protein 2	LKDQASVGAPAPK	G	1.8E-06
Q5F696	Enoyl-[acyl-carrier-protein] reductase [NADH]	EAFNTAHEISAYSLPALAK	A	0.000045

Q5F696	Enoyl-[acyl-carrier-protein] reductase [NADH]	TLAASGIADFGK	L	0.023
Q5F8I5	Genome-derived Neisseria antigen 1220	AMQAQITAER	E	0.000084
Q5F8I5	Genome-derived Neisseria antigen 1220	AMQAQITAEREK	R	0.00027
Q5F8I5	Genome-derived Neisseria antigen 1220	IAESEGRKIEQINLASGQR	E	0.0047
Q5F8I5	Genome-derived Neisseria antigen 1220	KIEQINLASGQR	E	2.2E-06
Q5F8I5	Genome-derived Neisseria antigen 1220	IEQINLASGQR	E	0.039
Q5F8I5	Genome-derived Neisseria antigen 1220	EAEIQQSEGEAQAAVNASNAEK	I	1E-07
Q5F8I5	Genome-derived Neisseria antigen 1220	QIAAALQTQGGADAVNLK	I	6.8E-12
Q5F8I5	Genome-derived Neisseria antigen 1220	IAEQYVAAFNNLAK	E	0.000012
Q5F731	Glutamate dehydrogenase	TDLNTLFANLK	Q	1.4E-06
Q5F731	Glutamate dehydrogenase	TDLNTLFANLKQR	N	0.00023
Q5F731	Glutamate dehydrogenase	NPKYTQQSLLER	I	0.0057
Q5F731	Glutamate dehydrogenase	YTQQSLLER	I	9.6E-06
Q5F731	Glutamate dehydrogenase	FLAFEQVFK	N	0.043
Q5F731	Glutamate dehydrogenase	VLISGSGNVAQYAAEK	A	7.1E-09
Q5F731	Glutamate dehydrogenase	ASNAGGVATSGLEMSQNAIR	L	3E-11
Q5F731	Glutamate dehydrogenase	VGDKVNYVNGANIAGFVK	V	2.1E-06
Q5F6F9	Glutamine synthetase	GGYAPVAPIDCGQDLR	S	0.00027
Q5F6F9	Glutamine synthetase	FATLVKR	A	0.0026
Q5F6F9	Glutamine synthetase	ALNAITNPSTNSYK	R	0.0074
Q5F6F9	Glutamine synthetase	ALNAITNPSTNSYKR	L	0.00001
Q5F6F9	Glutamine synthetase	LVPHFEAPTK	L	0.027
Q5F6F9	Glutamine synthetase	LVPHFEAPTKLAYSAK	N	0.0006
Q5F6F9	Glutamine synthetase	SASIRIPSVNSSK	A	0.0019
Q5F7G1	Glutathione synthetase	AADKVQTALK	E	0.015
Q5F7G1	Glutathione synthetase	FTAPTLVTTR	S	0.000026
Q5F7G1	Glutathione synthetase	YIPEIVHGDKR	I	0.046
Q5F7G1	Glutathione synthetase	ILIIGGEVVPYALAR	I	0.000095
Q5F8H4	Inosine-5'-monophosphate dehydrogenase	AYTFDDVLLVPAHSTVLPR	D	2.9E-07
Q5F8H4	Inosine-5'-monophosphate dehydrogenase	EITLNLPLLSAAMDTVTEAR	L	1.6E-07
Q5F8H4	Inosine-5'-monophosphate dehydrogenase	RHESGVVKDPVTVAPTTLIR	E	0.0025
Q5F8H4	Inosine-5'-monophosphate dehydrogenase	HESGVVKDPVTVAPTTLIR	E	0.000015
Q5F8H4	Inosine-5'-monophosphate dehydrogenase	TTEFPNANKDSEGR	L	0.0012

Q5F8H4	Inosine-5'-monophosphate dehydrogenase	VGAAVGTGGDTDERVK	A	0.00072
Q5F8H4	Inosine-5'-monophosphate dehydrogenase	IVAGVGVPQLTAIHNVAEALK	G	0.001
Q5F809	Lipid modified azurin protein	ACKEFTITLK	H	0.013
Q5F809	Lipid modified azurin protein	ASMGHNLVIAK	A	0.000086
Q5F809	Lipid modified azurin protein	LIGGGEESSLTLDPAK	L	2.2E-06
Q5F809	Lipid modified azurin protein	FACTFPGHGALMNGK	V	0.000017
Q5F809	Lipid modified azurin protein	FACTFPGHGALMNGKVTLVD	-	0.0016
Q5F501	Lipoprotein	DSAPAASAAAPSADNGAAK	K	0.0045
Q5F501	Lipoprotein	DSAPAASAAAPSADNGAAKK	E	0.000057
Q5F501	Lipoprotein	EIVFGTTVGDFGDMVK	E	0.00013
Q5F501	Lipoprotein	EQIQAELEKK	G	0.018
Q5F501	Lipoprotein	EHNLDITEAFQVPTAPLGLYPGK	L	0.00043
Q5F501	Lipoprotein	LKSLEEVKDGSTVSAPNDPSNFAR	A	5.9E-06
Q5F501	Lipoprotein	SLEEVKDGSTVSAPNDPSNFAR	A	0.000014
Q5F501	Lipoprotein	DGSTVSAPNDPSNFAR	A	0.0021
Q5F501	Lipoprotein	ALVMLNELGWIK	L	0.00013
Q5F501	Lipoprotein	LKDGINPLTASK	A	0.00014
Q5F501	Lipoprotein	IVELEAAQLPR	S	0.000075
Q5F501	Lipoprotein	LTEALFQEPSFAYVNWSAVK	T	2.8E-06
Q5F501	Lipoprotein	TADKDSQWLKDVTEAYNSDAFK	A	6.3E-07
Q5F501	Lipoprotein	RFEGYKYPAAWNEGAAK	-	5.7E-06
Q5F501	Lipoprotein	FEGYKYPAAWNEGAAK	-	0.000001
Q5F5V7	Major outer membrane protein porin P.IB	AGVQTYRSVEHTDGK	V	0.031
Q5F5V7	Major outer membrane protein porin P.IB	AGVQTYRSVEHTDGKVSK	V	0.007
Q5F5V7	Major outer membrane protein porin P.IB	AGVQTYRSVEHTDGKVSKVETGSEIADFGSK	I	2.2E-06
Q5F5V7	Major outer membrane protein porin P.IB	SVEHTDGKVSK	V	0.00007
Q5F5V7	Major outer membrane protein porin P.IB	SVEHTDGKVSKVETGSEIADFGSK	I	3.3E-06
Q5F5V7	Major outer membrane protein porin P.IB	VSKVETGSEIADFGSK	I	1.6E-07
Q5F5V7	Major outer membrane protein porin P.IB	VETGSEIADFGSK	I	1.2E-07
Q5F5V7	Major outer membrane protein porin P.IB	IGFKGQEDLGNGLK	A	7.8E-06
Q5F5V7	Major outer membrane protein porin P.IB	GQEDLGNGLK	A	0.000058
Q5F5V7	Major outer membrane protein porin P.IB	AVWQLEQGASVAGTNTGWGNK	Q	3E-10
Q5F5V7	Major outer membrane protein porin P.IB	AVWQLEQGASVAGTNTGWGNKQSFVGLK	G	0.03
Q5F5V7	Major outer membrane protein porin P.IB	QSFVGLKGGFGTIR	A	0.000011
Q5F5V7	Major outer membrane protein porin P.IB	AGSLNSPLK	N	0.00083
Q5F5V7	Major outer membrane protein porin P.IB	FTGNVLEISGMAQR	E	0.00055

Q5F5V7	Major outer membrane protein porin P.IB	YDSPEFAGFSGSVQYAPK	D	1.2E-09
Q5F5V7	Major outer membrane protein porin P.IB	LVGGYDNNALYVSVAAQQQDAK	L	6.2E-11
Q5F5V7	Major outer membrane protein porin P.IB	VSYAHGFK	G	0.000027
Q5F5V7	Major outer membrane protein porin P.IB	GTVDSANHDNTYDQVVVGAEYDFSKR	T	0.00001
Q5F5V7	Major outer membrane protein porin P.IB	RTSALVSAGWLQEGK	G	3.2E-08
Q5F5V7	Major outer membrane protein porin P.IB	TSALVSAGWLQEGK	G	2.1E-06
Q5F5V7	Major outer membrane protein porin P.IB	TSALVSAGWLQEGKGADK	I	8.9E-07
Q5F5V7	Major outer membrane protein porin P.IB	TSALVSAGWLQEGKGADKIVSTASAVVLR	H	0.000006
Q5F5V7	Major outer membrane protein porin P.IB	GADKIVSTASAVVLR	H	2E-07
Q5F5V7	Major outer membrane protein porin P.IB	IVSTASAVVLR	H	3.4E-06
Q5F4X7	Malonyl CoA-acyl carrier protein transacylase	TVEIKQPQIR	V	0.018
Q5F4X7	Malonyl CoA-acyl carrier protein transacylase	VIHNADVAAYDDAGK	I	0.000055
Q5F4X7	Malonyl CoA-acyl carrier protein transacylase	IKDALVR	Q	0.023
Q5F726	Multidrug efflux pump channel protein	NNLLPTLAANANGSR	Q	0.0037
Q5F726	Multidrug efflux pump channel protein	AGVISAVALRQQEALIESAK	A	1.4E-07
Q5F726	Multidrug efflux pump channel protein	NALATLINRPIPEDLPAGLPLDK	Q	0.002
Q5F726	Multidrug efflux pump channel protein	LPAGLSSEVLLDRPDIR	A	0.000051
Q5F726	Multidrug efflux pump channel protein	AAEHALKQANANIGAAR	A	9.1E-06
Q5F726	Multidrug efflux pump channel protein	QANANIGAAR	A	0.0023
Q5F726	Multidrug efflux pump channel protein	LTGSVGTGSVELGGLFK	S	4.4E-06
Q5F726	Multidrug efflux pump channel protein	YKHGVSGALDLLDAER	I	1.6E-06
Q5F6N6	Outer membrane opacity protein D	NKISTVSDYFR	N	0.0017
Q5F6N6	Outer membrane opacity protein D	ISTVSDYFR	N	0.00018
Q5F6N6	Outer membrane opacity protein D	VSVGYDFGGWR	I	8.5E-06
Q5F6N6	Outer membrane opacity protein D	VAYGHVR	H	0.0018
Q5F5W8	Outer membrane protein assembly factor BamA	TEPSTVFNYLPVK	V	0.00035
Q5F5W8	Outer membrane protein assembly factor BamA	VGDTYNDTHGSAIIK	S	0.0051
Q5F5W8	Outer membrane protein assembly factor BamA	GKLNIQITPK	V	0.003

Q5F5W8	Outer membrane protein assembly factor BamA	ILDTDIQTNEDKTR	Q	0.00024
Q5F5W8	Outer membrane protein assembly factor BamA	QQMTAVLGEIQNR	M	0.017
Q5F5W8	Outer membrane protein assembly factor BamA	IYVNEIHITGNNK	T	0.000011
Q5F5W8	Outer membrane protein assembly factor BamA	TKEIPFFENFYGGGLGSVR	G	0.000019
Q5F9W0	Outer membrane protein assembly factor BamD	GLVLFNEDQSFLNK	L	7.4E-06
Q5F9W0	Outer membrane protein assembly factor BamD	GAYIAAANR	A	0.0006
Q5F5Y8	Outer membrane protein assembly factor BamE	AVAALRPGMTKDQVLLLLGSPILR	D	0.0033
Q5F6I1	Outer membrane protein PIII	NAYFDKASQGR	V	0.000056
Q5F6I1	Outer membrane protein PIII	ASQGRVECGDAVAVPEPEPAPVAVVEQAPQYVDETISLSAK	T	0.00018
Q5F6I1	Outer membrane protein PIII	TLFGFDKDSLR	A	0.01
Q5F6I1	Outer membrane protein PIII	TLFGFDKDSLRAEAQDNLK	V	0.000053
Q5F6I1	Outer membrane protein PIII	DSLRAEAQDNLK	V	0.00027
Q5F6I1	Outer membrane protein PIII	TNVQSVR	V	0.00031
Q5F6I1	Outer membrane protein PIII	VEGHTDFMGSEK	Y	0.0013
Q5F6I1	Outer membrane protein PIII	VEGHTDFMGSEKYNQALSER	R	3.9E-07
Q5F6I1	Outer membrane protein PIII	YNQALSER	R	0.000018
Q5F6I1	Outer membrane protein PIII	RAYVVANNLVSNGVPASR	I	4.1E-07
Q5F6I1	Outer membrane protein PIII	AYVVANNLVSNGVPASR	I	2.5E-09
Q5F6I1	Outer membrane protein PIII	ISAVGLGESQAQMTQVCQAEVAK	L	5.7E-09
Q5F6I1	Outer membrane protein PIII	EALIACIEPDR	R	0.0011
Q5F6I1	Outer membrane protein PIII	EALIACIEPDRRVDVK	I	0.044
Q5F6I1	Outer membrane protein PIII	IRSIVTR	Q	0.02
Q5F7F3	Peptidyl-prolyl cis-trans isomerase	SLKQMKEQGAEIDLK	V	0.003
Q5F7F3	Peptidyl-prolyl cis-trans isomerase	FLQEQQAK	A	0.027
Q5F7F3	Peptidyl-prolyl cis-trans isomerase	ANKEKGEAFLKENAAK	D	0.000039
Q5F7F3	Peptidyl-prolyl cis-trans isomerase	ANKEKGEAFLKENAAKDGVK	T	0.046
Q5F7F3	Peptidyl-prolyl cis-trans isomerase	DGVKTTASGLQYK	I	0.03
Q5F7F3	Peptidyl-prolyl cis-trans isomerase	DGVKTTASGLQYKITK	Q	0.00013

Q5F7F3	Peptidyl-prolyl cis-trans isomerase	TTASGLQYK	I	0.0036
Q5F7F3	Peptidyl-prolyl cis-trans isomerase	TTASGLQYKITK	Q	0.000073
Q5F7F3	Peptidyl-prolyl cis-trans isomerase	QGEGKQPTKDDIVTVEYEGR	L	0.0017
Q5F7F3	Peptidyl-prolyl cis-trans isomerase	LIDGTVFDSSK	A	0.0018
Q5F7F3	Peptidyl-prolyl cis-trans isomerase	ANGGPATFPLSQVIPGWTEGVR	L	1E-07
Q5F7F3	Peptidyl-prolyl cis-trans isomerase	LLKEGGEATFYIPSNLAYREQGAGEK	I	0.00024
Q5F7F3	Peptidyl-prolyl cis-trans isomerase	EGGEATFYIPSNLAYR	E	0.000011
Q5F7F3	Peptidyl-prolyl cis-trans isomerase	IGPNATLVFDVK	L	2.6E-06
Q5F7F3	Peptidyl-prolyl cis-trans isomerase	IGAPENAPAKQPDQVDIK	K	0.0023
Q5F7F3	Peptidyl-prolyl cis-trans isomerase	IGAPENAPAKQPDQVDIKK	V	0.0044
Q5F7F3	Peptidyl-prolyl cis-trans isomerase	IGAPENAPAKQPDQVDIKKVN	-	0.0013
Q5F820	Peptidyl-prolyl cis-trans isomerase	FKATVESVR	D	0.0068
Q5F9L7	Peptidyl-prolyl cis-trans isomerase	TVVQDWGYAVFGK	V	0.000085
Q5F9L7	Peptidyl-prolyl cis-trans isomerase	VVDGFDVVDAIESVsTKR	H	1.7E-08
Q5F668	Phospho-2-dehydro-3-deoxyheptonate aldolase	ELLPPIAHLYELPISK	E	0.013
Q5F668	Phospho-2-dehydro-3-deoxyheptonate aldolase	EASGLVHR	T	0.013
Q5FA34	Phosphoenolpyruvate synthase	ADNYVIWFENLR	M	0.000016
Q5FA34	Phosphoenolpyruvate synthase	NASLGEMISQLTEK	G	0.000026
Q5FA34	Phosphoenolpyruvate synthase	VPGGFATTADAYR	A	9.6E-07
Q5FA34	Phosphoenolpyruvate synthase	AFLAHNGLNER	I	0.0033
Q5FA34	Phosphoenolpyruvate synthase	VHKGFEHDIVALSAGVQR	M	2.1E-08
Q5FA34	Phosphoenolpyruvate synthase	GFEHDIVALSAGVQR	M	0.000035
Q5FA34	Phosphoenolpyruvate synthase	MIFTDKAEAGK	S	0.0078
Q5FA34	Phosphoenolpyruvate synthase	DGLDGKLYILQARPETVK	S	0.00013
Q5FA34	Phosphoenolpyruvate synthase	LYILQARPETVK	S	0.000032
Q5FA34	Phosphoenolpyruvate synthase	VRDEMGLTNVEIMIPFVR	T	0.00066
Q5FA34	Phosphoenolpyruvate synthase	ALKENGLER	G	0.0052
Q5FAD1	Pilin assembly protein	KAELLLNSSDKNTEQAAAPAAEQN	-	0.013
Q5F693	Pilus assembly protein	KITQEDITVFTR	Q	0.000012
Q5F693	Pilus assembly protein	AGLPLMQAFEIVAR	G	5.4E-08
Q5F693	Pilus assembly protein	GQVEQGSSLSR	A	0.00045
Q5FAD0	Pilus assembly protein	SIDAASLNNLRDELASIR	S	0.013
Q5FAD0	Pilus assembly protein	IAQSPENGGNPDGK	S	0.035
Q5FAD0	Pilus assembly protein	SSILNLSAIATTYQAK	S	8.6E-08

Q5FAG7	Pilus-associated protein	VQMYSASVsTYPGSSSSR	I	3.1E-07
Q5FAG7	Pilus-associated protein	KDIEGNDSDLAK	E	0.0078
Q5FAG7	Pilus-associated protein	TQNGKYAAFLASGYAAK	N	0.000033
Q5FAG7	Pilus-associated protein	YAAFLASGYAAK	N	0.000023
Q5FAG7	Pilus-associated protein	TIFEGDKPITSAPAVSR	L	0.00017
Q5FAG7	Pilus-associated protein	TVCPNGYVYDKPVNVR	Y	0.00071
Q5F7F1	Probable cytosol aminopeptidase	FSQAVFHSAHEAAVK	E	0.0023
Q5F7F1	Probable cytosol aminopeptidase	VAEAQVYGQSLCR	D	0.036
Q5F7F1	Probable cytosol aminopeptidase	TAKAEAEKLGAHAK	I	1.2E-06
Q5F7F1	Probable cytosol aminopeptidase	GATGRPVPLLMNYLR	N	0.00039
Q5FA43	Protein translocase subunit SecD	QNITTLHNR	V	0.0002
Q5F8V3	Pseudouridine synthase	QWRDGAAPSAKK	T	0.048
Q5F8V3	Pseudouridine synthase	GQFYELNPAEVANILK	W	9.5E-07
Q5F874	Putative 2-oxoglutarate dehydrogenase, E1 component	IASAVAGGADEAMLKK	Q	4.8E-07
Q5F874	Putative 2-oxoglutarate dehydrogenase, E1 component	IQGVGAAQLDPLK	R	2.6E-06
Q5F874	Putative 2-oxoglutarate dehydrogenase, E1 component	IQGVGAAQLDPLKR	I	0.000025
Q5F874	Putative 2-oxoglutarate dehydrogenase, E1 component	NYFESVLSTPHYNADQK	R	2.1E-06
Q5F874	Putative 2-oxoglutarate dehydrogenase, E1 component	ILKEMTAAETLER	Y	0.0036
Q5F874	Putative 2-oxoglutarate dehydrogenase, E1 component	STVHCTDIAK	M	0.015
Q5F874	Putative 2-oxoglutarate dehydrogenase, E1 component	EKIETGLPAADIER	L	0.00033
Q5F874	Putative 2-oxoglutarate dehydrogenase, E1 component	LTEKFTAVPEGFALHPTAK	R	0.00019
Q5F874	Putative 2-oxoglutarate dehydrogenase, E1 component	FTAVPEGFALHPTAK	R	1.3E-07
Q5F874	Putative 2-oxoglutarate dehydrogenase, E1 component	QAIDWGMAETLAYASLLTK	G	1.1E-09
Q5F874	Putative 2-oxoglutarate dehydrogenase, E1 component	VVLCAGQVYYDLEAGR	A	1.1E-06
Q5F874	Putative 2-oxoglutarate dehydrogenase, E1 component	VEQLYPFPYDEVKAELAK	Y	0.011
Q5F874	Putative 2-oxoglutarate dehydrogenase, E1 component	NQGAFYQIR	H	0.0022
Q5F874	Putative 2-oxoglutarate dehydrogenase, E1 component	HRIEDVISEEQK	L	0.038

Q5F4Y0	Putative 3-oxoacyl-[acyl-carrier protein] reductase	IALVTGASR	G	0.01
Q5F4Y0	Putative 3-oxoacyl-[acyl-carrier protein] reductase	GIGAAIADTLAAAGAK	I	7E-08
Q5F4Y0	Putative 3-oxoacyl-[acyl-carrier protein] reductase	IIGTATGESGAAAISK	R	1.2E-06
Q5F4Y0	Putative 3-oxoacyl-[acyl-carrier protein] reductase	IIGTATGESGAAAISKR	L	0.033
Q5F4Y0	Putative 3-oxoacyl-[acyl-carrier protein] reductase	LAQWGGEGR	V	0.00026
Q5F4Y0	Putative 3-oxoacyl-[acyl-carrier protein] reductase	VLNSAEPETVENLIADIEK	T	5.5E-07
Q5F4Y0	Putative 3-oxoacyl-[acyl-carrier protein] reductase	TFGKLDILVNNAGITR	D	0.00021
Q5F4Y0	Putative 3-oxoacyl-[acyl-carrier protein] reductase	LDILVNNAGITR	D	0.033
Q5F4Y0	Putative 3-oxoacyl-[acyl-carrier protein] reductase	ALPEETRQTFTAQTALGR	F	0.019
Q5F4Y0	Putative 3-oxoacyl-[acyl-carrier protein] reductase	FGDAQDIADAVLFLASDQAK	Y	4.7E-12
Q5F4Y0	Putative 3-oxoacyl-[acyl-carrier protein] reductase	YITGQTLHVNGGMLMP	-	0.00071
Q5F6I5	Putative ABC transporter, ATP-binding protein	LGNEVIEFVNVSK	S	3.5E-07
Q5F6I5	Putative ABC transporter, ATP-binding protein	VPAGAIVGIIGPNGAGK	S	9.7E-07
Q5F6I5	Putative ABC transporter, ATP-binding protein	DILQVGQFEIPAR	Q	0.000018
Q5F9W7	Putative ABC transporter, ATP-binding protein, amino acid	KGEVVVVCGPSGSGK	S	0.037
Q5F9W7	Putative ABC transporter, ATP-binding protein, amino acid	DAFPSQLSGGQQQR	V	0.00063
Q5F9W7	Putative ABC transporter, ATP-binding protein, amino acid	AKQFLQQVMTH	-	0.017
Q5F9M1	Putative ABC transporter, periplasmic binding protein, amino acid	INNKGTVTVGTEGTYAPFTYHDKDGK	L	0.000029
Q5F9M1	Putative ABC transporter, periplasmic binding protein, amino acid	INNKGTVTVGTEGTYAPFTYHDKDGKLTGYDVEVTR	A	0.000055
Q5F9M1	Putative ABC transporter,	VEFKETQWDSMMAGLK	A	0.000062

	periplasmic binding protein, amino acid			
Q5F9M1	Putative ABC transporter, periplasmic binding protein, amino acid	AGRFDVVANQVGLTSPER	Q	2.4E-08
Q5F9M1	Putative ABC transporter, periplasmic binding protein, amino acid	FDVVANQVGLTSPER	Q	2.5E-06
Q5F9M1	Putative ABC transporter, periplasmic binding protein, amino acid	QATFDKSEPYSWSGAVLVAHNDSNIK	S	0.00001 5
Q5F9M1	Putative ABC transporter, periplasmic binding protein, amino acid	SEPYSWSGAVLVAHNDSNIK	S	0.00001 1
Q5F9M1	Putative ABC transporter, periplasmic binding protein, amino acid	TAQSLTSNYGEK	A	0.00003 1
Q5F9M1	Putative ABC transporter, periplasmic binding protein, amino acid	AKAAGAQLVPVDGLAQSLTLIEQK	R	0.00016
Q5F9M1	Putative ABC transporter, periplasmic binding protein, amino acid	AAGAQLVPVDGLAQSLTLIEQK	R	1.1E-08
Q5F9M1	Putative ABC transporter, periplasmic binding protein, amino acid	AAGAQLVPVDGLAQSLTLIEQKR	A	5.8E-09
Q5F9M1	Putative ABC transporter, periplasmic binding protein, amino acid	RADATLNDELAVLDYLK	K	3.6E-08
Q5F9M1	Putative ABC transporter, periplasmic binding protein, amino acid	RADATLNDELAVLDYLKK	N	5.7E-08
Q5F9M1	Putative ABC transporter, periplasmic binding protein, amino acid	ADATLNDELAVLDYLK	K	4.1E-08
Q5F9M1	Putative ABC transporter, periplasmic binding protein, amino acid	ADATLNDELAVLDYLKK	N	0.00013
Q5F9M1	Putative ABC transporter, periplasmic binding protein, amino acid	IVWSAPADEKVGSGLIVNK	G	5.2E-08
Q5F9M1	Putative ABC transporter, periplasmic binding protein, amino acid	IVWSAPADEKVGSGLIVNKGNDEAVAK	F	0.00019
Q5F9M1	Putative ABC transporter, periplasmic binding protein, amino acid	VGSGLIVNK	G	0.033
Q5F9M1	Putative ABC transporter,	VGSGLIVNKGNDEAVAK	F	0.00004

	periplasmic binding protein, amino acid			
Q5F9M1	Putative ABC transporter, periplasmic binding protein, amino acid	FSTAINELK	A	0.00009 9
Q5F9M1	Putative ABC transporter, periplasmic binding protein, amino acid	FSTAINELKADGTLK	K	4.2E-06
Q5F9M1	Putative ABC transporter, periplasmic binding protein, amino acid	FSTAINELKADGTLKK	L	0.00003 1
Q5F9M1	Putative ABC transporter, periplasmic binding protein, amino acid	KLGEQFFGKDISVQ	-	3.4E-07
Q5F9M1	Putative ABC transporter, periplasmic binding protein, amino acid	LGEQFFGKDISVQ	-	0.00028
Q5F6Q5	Putative ABC transporter, periplasmic binding protein, polyamine	SGYDLVVPGIAFLPR	Q	2.6E-06
Q5F6Q5	Putative ABC transporter, periplasmic binding protein, polyamine	VNKDLIPNYKNIDPELLK	M	0.015
Q5F6Q5	Putative ABC transporter, periplasmic binding protein, polyamine	GSNPEDLKAAAEVLK	S	0.00011
Q5F6Q5	Putative ABC transporter, periplasmic binding protein, polyamine	RFSPSIIDELAR	G	0.017
Q5F6Q5	Putative ABC transporter, periplasmic binding protein, polyamine	FSPSIIDELAR	G	0.00083
Q5F6Q5	Putative ABC transporter, periplasmic binding protein, polyamine	ARSEEVKNNVGIEVLTPK	G	4.8E-06
Q5F6Q5	Putative ABC transporter, periplasmic binding protein, polyamine	SEEVKNNVGIEVLTPK	G	0.00004 8
Q5F6Q5	Putative ABC transporter, periplasmic binding protein, polyamine	YINYTLDPEIAAK	N	0.00074
Q5F6Q5	Putative ABC transporter, periplasmic binding protein, polyamine	NGIAVTFAPASKPAR	E	0.00004 4
Q5F6Q5	Putative ABC transporter, periplasmic binding protein, polyamine	EKMPAELVNTR	S	0.00041
Q5F6Q5	Putative ABC transporter,	MPAELVNTR	S	0.028

	periplasmic binding protein, polyamine			
Q5F7C5	Putative ABC transporter, periplasmic binding protein, polyamine	NGNFVTYAPSSKPAR	D	0.0064
Q5FA28	Putative ABC transporter, periplasmic binding protein, polyamine	SGYDLTAPSIANVGR	Q	3.1E-06
Q5FA28	Putative ABC transporter, periplasmic binding protein, polyamine	AQIPHYGNIDKDLLK	M	0.00022
Q5FA28	Putative ABC transporter, periplasmic binding protein, polyamine	TGVGVWVDSFMIPR	D	0.00078
Q5FA28	Putative ABC transporter, periplasmic binding protein, polyamine	YIDYTLRPEVAAK	N	0.0039
Q5F8K8	Putative ABC-transporter, ATP-binding protein	FAEYDGYTAEAR	A	1.6E-07
Q5F8K8	Putative ABC-transporter, ATP-binding protein	LQELQEFVAR	F	0.025
Q5F8K8	Putative ABC-transporter, ATP-binding protein	LKQADKIKSEMVEVKPSTR	Q	0.008
Q5F8K8	Putative ABC-transporter, ATP-binding protein	SEMVEVKPSTR	Q	0.014
Q5F8K8	Putative ABC-transporter, ATP-binding protein	LAIIGPNGAGK	S	0.00068
Q5F8B6	Putative ABC-type transporter, ATP-binding protein	SREDKIQTASAPK	A	0.013
Q5F655	Putative adenylosuccinate lyase	LLELTPALYVGK	A	0.0037
Q5F655	Putative adenylosuccinate lyase	LLELTPALYVGKAEALAK	R	0.000037
Q5F6V3	Putative alcohol dehydrogenase	MKMQAVVVNK	N	0.00014
Q5F6V3	Putative alcohol dehydrogenase	ETGADLVVNAAKEDAAK	V	0.00043
Q5F6V3	Putative alcohol dehydrogenase	TGGAHAAVVTAVSAAAFNSAVNCVR	A	0.015
Q5F6V3	Putative alcohol dehydrogenase	VVAVGLPPESMDLSIPR	L	0.033
Q5F6V3	Putative alcohol dehydrogenase	LVLDGIEVVGSLVGTR	K	2.1E-07
Q5F6V3	Putative alcohol dehydrogenase	LVLDGIEVVGSLVGTRK	D	5.8E-07
Q5F6V3	Putative alcohol dehydrogenase	ALDEAPAIFQEMR	E	0.000049
Q5F8U5	Putative aldehyde dehydrogenase	ILAAHAGANLKK	T	0.00042
Q5F758	Putative amino acid aminotransferase	IVTVQTLGGSGALK	V	0.0036
Q5F6U0	Putative aminopeptidase	LIEGINPSTFFK	S	0.00038

Q5F6U0	Putative aminopeptidase	VVPVGTPTAEQKR	D	0.0049
Q5F6U0	Putative aminopeptidase	RLEPLTEGAAK	A	0.004
Q5FA72	Putative aminopeptidase N	YLKDYQTPAYR	I	0.013
Q5FA72	Putative aminopeptidase N	LKNNVFELTIK	Q	0.02
Q5FA72	Putative aminopeptidase N	RSDTLQQVQTALQHPK	F	1.7E-06
Q5FA72	Putative aminopeptidase N	NVPHFHAQDGSGYR	F	0.0089
Q5F9D9	Putative aminotransferases	TYHGSAMSIPVQR	A	0.0084
Q5F9D9	Putative aminotransferases	AAIQVLPGR	F	0.0011
Q5F6R0	Putative aspartyl-tRNA synthetase	LRYQVATGVR	R	0.0083
Q5F6R0	Putative aspartyl-tRNA synthetase	VHPGEFFALPQSPQLFK	Q	0.00027
Q5F6R0	Putative aspartyl-tRNA synthetase	QVFKDALNVDLGDFPR	M	0.000001
Q5F6R0	Putative aspartyl-tRNA synthetase	VVALRVPNGAK	F	0.0008
Q5F6R0	Putative aspartyl-tRNA synthetase	VNDAGNLSNGEDSGLQSPIVK	F	0.00049
Q5F6R0	Putative aspartyl-tRNA synthetase	YVAVHHPFTAPK	E	0.026
Q5F6R0	Putative aspartyl-tRNA synthetase	EGHEDLMVSDPANCLAR	A	0.002
Q5F6R0	Putative aspartyl-tRNA synthetase	FGFLLDNLK	F	0.00046
Q5F6R0	Putative aspartyl-tRNA synthetase	FGAPPHGGLAFGLDR	L	0.00014
Q5F6R0	Putative aspartyl-tRNA synthetase	LVTLMTGAESIRDVIAFPK	T	0.039
Q5F6R0	Putative aspartyl-tRNA synthetase	AQCLLTDAPNSVDDK	Q	8.8E-07
Q5F6R0	Putative aspartyl-tRNA synthetase	AQCLLTDAPNSVDDKQLR	E	0.017
Q5FAG6	Putative atpase	TLLDTVAVPNTAR	T	0.0022
Q5FAG6	Putative atpase	LNVPLLGQLPLSLPVR	E	0.000068
Q5F9I6	Putative ATP-dependent C1p protease	QKKVIGK	A	0.00085
Q5F8W0	Putative ATP-dependent RNA helicase	GIDVPTITHVINYDLPK	Q	0.0029
Q5F8W0	Putative ATP-dependent RNA helicase	RGDHKPGKEGFGGK	T	0.00087
Q5FA80	Putative ATP-dependent RNA helicase	ALAGHDLLAAAQTGTGK	T	3.9E-07
Q5FA80	Putative ATP-dependent RNA helicase	YATASTSPAMHPVR	M	0.0004
Q5F697	Putative branched-chain amino acid aminotranferase	IVLFRPTANIAR	M	0.00094
Q5F697	Putative branched-chain amino acid aminotranferase	AADEIPDAPAALYLRPTLIGTDPVIGK	A	0.0029

Q5F697	Putative branched-chain amino acid aminotranferase	AGSPSETALLYILASPVGDYFK	V	0.000026
Q5FAB9	Putative carboxypeptidase, penicillin binding protein	RSDNLIAR	S	0.025
Q5F932	Putative carboxy-terminal processing protease	AVIVGTQSFGK	G	0.0029
Q5F8D2	Putative cell-division protein	SPDVSQGQSVsDGTAVR	D	0.00013
Q5F8D2	Putative cell-division protein	APHLLVAGTTGSGK	S	0.000059
Q5F4Z9	Putative chromosome segregation protein	AAQIAGLSEIPAVIK	T	0.0031
Q5F4Z9	Putative chromosome segregation protein	TISDETALAMGLIENLQR	E	3.7E-06
Q5F4Z9	Putative chromosome segregation protein	LADEFGLTHETIAQAVGK	S	8.8E-09
Q5F4Z9	Putative chromosome segregation protein	LLSLPESVQEMLYQR	R	0.000003
Q5F4Z9	Putative chromosome segregation protein	ALLTLPVVEQLELAQK	A	0.0019
Q5F4Z9	Putative chromosome segregation protein	SQAALQNKRPEPK	K	0.0007
Q5F7W9	Putative ClpB protein	QRLQQHLNSLPK	V	0.032
Q5F7W9	Putative ClpB protein	VSGQQGGEILPSR	E	0.0015
Q5F7W9	Putative ClpB protein	EAGATEQNINAAIDAVR	G	0.00011
Q5F7W9	Putative ClpB protein	TKNNPVLIGEPGVGK	T	0.00057
Q5F7W9	Putative ClpB protein	LLVLDLAALIAGAK	Y	3E-09
Q5F7W9	Putative ClpB protein	VLVGEPSVEDTIAILR	G	2.1E-07
Q5F7W9	Putative ClpB protein	FLPDKAIDLIDEAASR	V	0.0002
Q5F7W9	Putative ClpB protein	AISDGAANIKK	Q	0.0029
Q5F7W9	Putative ClpB protein	IKIEQAKR	Q	0.05
Q5F7W9	Putative ClpB protein	NNVGAEEIAEVVSR	M	3.2E-06
Q5F7W9	Putative ClpB protein	SGLADPNKPYGSFLFLGPTGVGK	T	0.004
Q5F7W9	Putative ClpB protein	ALAGFLFDSEDHLIR	I	0.000012
Q5F7W9	Putative ClpB protein	AIQSEIENPLAK	A	0.0011
Q5F9I4	Putative cold shock protein	GFGFITPDEGGEDLFAHFSAINMEGFK	T	0.000079
Q5F9I4	Putative cold shock protein	GFGFITPDEGGEDLFAHFSAINMEGFKTLK	E	0.0032
Q5F9I4	Putative cold shock protein	VSFDVTTGPK	G	0.0019
Q5F9I4	Putative cold shock protein	GKQAANIQAA	-	0.018
Q5F759	Putative cytochrome	IQPVGQLTMGDGIPVGER	Q	1.9E-07
Q5F759	Putative cytochrome	GGAADLTDQELKR	A	0.0011
Q5F759	Putative cytochrome	AEDKGAAAPAVGVDGK	K	0.00017
Q5F759	Putative cytochrome	AEDKGAAAPAVGVDGKK	V	0.0013
Q5F759	Putative cytochrome	HALEGFNAMPAK	G	0.0092
Q5FAC5	Putative cytochrome	SGEANPKENPELGAK	I	0.0001
Q5FAC5	Putative cytochrome	MSEEDLKAVANFIQGLR	-	2.5E-07
Q5F716	Putative cytochrome c oxidase subunit	AATQPAPGVKPYNALQVAGR	D	0.000023
